# Comparing methods of category learning: Classification versus feature inference

**DOI:** 10.3758/s13421-020-01022-8

**Published:** 2020-02-20

**Authors:** Emma L. Morgan, Mark K. Johansen

**Affiliations:** School of Psychology, Cardiff University, Tower Building, Park Place, Cardiff, Wales CF10 3AS UK

**Keywords:** Feature inference, Classification, Categorization, Category learning, Rule representation

## Abstract

Categories have at least two main functions: classification of instances and feature inference. Classification involves assigning an instance to a category, and feature inference involves predicting a feature for a category instance. Correspondingly, categories can be learned in two distinct ways, by classification and feature inference. A typical difference between these in the perceptual category learning paradigm is the presence of the category label as part of the stimulus in feature inference learning and not in classification learning. So we hypothesized a label-induced rule-bias in feature inference learning compared to classification and evaluated it on an important starting point in the field for category learning – the category structures from Shepard, Hovland, and Jenkins (Psychological Monographs: General and Applied, 75(13), 1-42, 1961). They classically found that classification learning of structures consistent with more complex rules resulted in poorer learning. We compared feature inference learning of these structures with classification learning and found differences between the learning tasks supporting the label-bias hypothesis in terms of an emphasis on label-based rules in feature inference. Importantly, participants’ self-reported rules were largely consistent with their task performance and indicated the preponderance of rule representation in both tasks. So, while the results do not support a difference in the kind of representation for the two learning tasks, the presence of category labels in feature inference tended to focus rule formation. The results also highlight the specialized nature of the classic Shepard et al. (1961) stimuli in terms of being especially conducive to the formation of compact verbal rules.

## Introduction

Making feature inferences about instances of categories is a crucial cognitive ability in daily life. When presented with a novel instance from a known category, people can infer its properties and interact with it. For example, correctly categorizing an apple allows the inference of edibility.

There are at least two ways to learn about categories via feedback: One is by classification learning, assigning an instance to a category and then being told the correct category, for example, classifying a small, furry animal as a cat, not a dog. Another is by feature inference learning, inferring features of known category instances and being told the correct feature, for example, inferring a cat is likely to purr if you pet it (rather than bite). A key difference between these two learning tasks is the presence of the category membership information as essentially part of the stimulus in feature inference. In perceptual category learning, this category information commonly takes the form of a category label. This difference in available information suggests the possibility that classification and feature inference learning result in fundamentally different category representations and decision making because of the presence of the label in feature inference.

Consistent with this, Yamauchi and Markman (1998) hypothesized that feature inference learning tasks encourage learning the internal structure of each category and the typicality of individual features within a category, which induces prototype representation. Classification learning, in contrast, encourages learning the differences between categories. Anderson, Ross, and Chin-Parker (2002) further supported Yamauchi and Markman’s hypothesis: Participants who had completed a feature-inference learning task performed better on single feature classifications than full instance classifications. This suggests that feature inference encourages the learning of prototypical features and thus prototype representation. Johansen and Kruschke (2005) also contrasted these kinds of learning on the “5-4” category structure from Medin and Schaffer (1978) and argued that feature inference encouraged the formation of a set of label-based rules that sometimes mimicked prototypes and sometimes didn’t. From this they suggested that feature inference learning does not induce a prototype representation per se but rather a tendency to form a representation based on the category labels, in contrast to classification learning.

Similar to learning, decision making for classification versus feature inference has been argued to be different, with feature inference especially influenced by category membership information. Gelman and Markman (1986) showed that feature inference decision making for real-world categories (e.g., birds) was more heavily influenced by category membership than by perceptual similarity. Correspondingly, in the perceptual category learning paradigm, Yamauchi and Markman (2000) found that feature inferences were more likely to be determined by a category label than by perceptual similarity. From this they argued for the special status of the category labels. This was replicated by Johansen, Savage, Fouquet, and Shanks (2015), but they argued that the effect was due to the perceptual salience of the category labels. Nevertheless, both of these suggest that the presence of the label in feature inference plausibly induces a difference in focus compared to classification.

Adapting the hypotheses from Yamauchi and Markman (1998, 2000) in light of Johansen and Kruschke (2005) and Johansen et al. (2015), we propose a label-induced rule-bias hypothesis: Category labels in feature inference bias participants to use the labels to try to form rules. In contrast, classification learning does not result in such a bias due to the lack of the category labels as part of the stimuli. Note that in contrast to the prior hypotheses, this is not a hypothesis about what representation participants *definitely* use but rather a bias for *trying* to use a representation based on the labels as elaborated below.

**Fig. 1:**
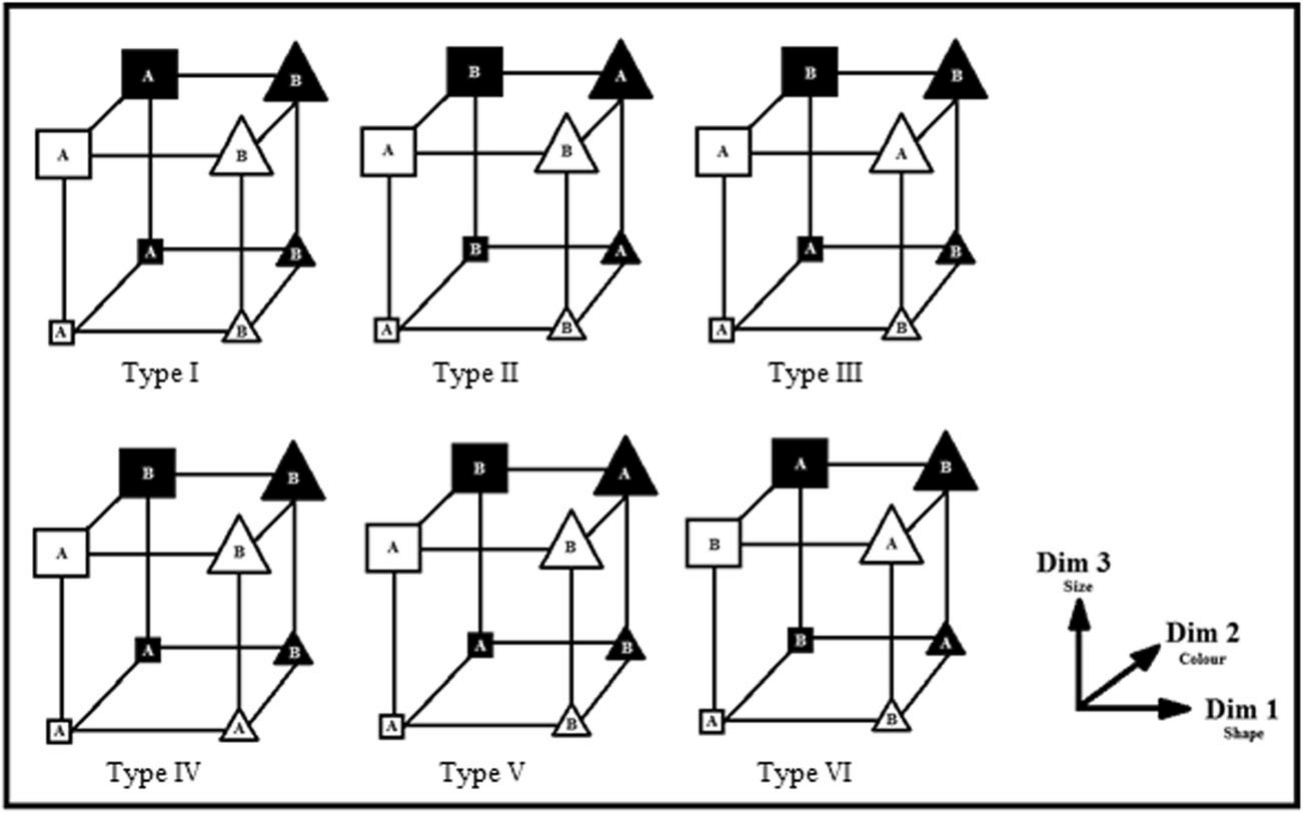
The six types of category structures from Shepard et al. (1961). The diagram in the bottom right shows the assignment of the three stimulus dimensions to each dimension of the abstract category structures, i.e., the cubes. Each corner of a cube represents a category instance as composed of a feature value from each of the three binary-valued stimulus dimensions. “A” labels indicate instances of one category and “B” labels indicate instances of the other category (adapted in part from Kruschke, 1992, and Shepard et al., 1961)

The purpose of this research was to go back to an important starting point for category learning – Shepard, Hovland, and Jenkins (1961) – and to re-evaluate these classic category structures in terms of feature inference learning to test the label-bias hypothesis. The conceptual reason these category structures are important is that they represent an evaluation of the learnability of what are among the simplest, non-trivial categories. Specifically, Shepard et al. evaluated the relative learnability of all possible category structures formed with eight instances, equally split into two categories, with instances composed of features from three binary-valued dimensions. There are six basic category structure types that are consistent with these constraints (see Fig. 1). In the figure, each type is a cube with the specific instances of the A and B categories at the corners of the cube and the edges indicating the three feature dimensions and thus the features composing each instance. The Type I structure can be learned using a rule on a single dimension, dimension one, that allows one feature to be exclusively associated with one category and the other feature to be exclusively associated with the other category, for example, the feature “square”-shaped only occurs in instances of category A, and the feature “triangle”-shaped in category B. The Type II structure, XOR, can be learned using a rule based on the configuration of the first two dimensions, for example, instances with features “white” and “square” or “black” and “triangle” are occurrences of category A while “black” and “square” or “white” and “triangle,” category B. For Types III, IV, and V, learning a rule on the first dimension allows correct categorization of six out of the eight instances, but the remaining two exceptions have to be handled in some other way, for example, for Type V, category A instances are either “square” or “large” “black” and “triangle” and category B either “triangle” or “large” “black” and “square.” Finally, for Type VI, each category instance can be memorized, or the structure learned in terms of the Odd-Even rule. The Odd-Even rule requires remembering a single instance and if another instance varies from that instance by one feature or all three features then the correct category is the opposite of the category for that instance. If the new instance varies by two features, then the correct category is the same as the remembered instance (Shepard et al., 1961).

Shepard et al.’s (1961) classic findings were systematic differences in the learning difficulty for these six category structures: The Type I category structure was the easiest to learn, Type II was more difficult, Types III, IV, and V were equally difficult but all harder than Type II, and Type VI was the most difficult; in summary I<II<III=IV=V<VI. This pattern of learning has been replicated and evaluated many times (Edmunds & Wills, 2016; Griffiths, Christian, & Kalish, 2008; Kruschke, 1992; Kurtz, 2007; Love, Medin, & Gureckis, 2004; Nosofsky, Gluck, Palmeri, McKinley, & Glauthier, 1994a; Nosofsky, Palmeri, & McKinley, 1994b; Rehder & Hoffman, 2005; Smith, Minda, & Washburn, 2004; Žauhar, Bajšanski, & Domijan, 2016) though some studies have not clearly differentiated the learning between some specific types (Kurtz, Levering, Stanton, Romero, & Morris 2013; Lewandowsky, 2011; Love, 2002; Žauhar, Bajšanski, & Domijan, 2014). Shepard et al.’s (1961) key conclusion was that this pattern of learning difficulty reflects the complexity of the rules that allow accurate performance, and as such these results clarify the cognitive mechanisms involved with basic category learning.

Nosofsky et al. (1994a) replicated Shepard et al. (1961) with similar stimuli and found the same ordering of the types, and this represents a kind of canonical replication. However, deviations from the standard type ordering have been found as a result of initial task instructions and the specific stimuli used: Nosofsky and Palmeri (1996) compared integral dimension stimuli to the classic separable dimension stimuli used by Shepard et al. (1961) and found that Type II was more difficult than Types III and IV and not significantly different from Type V. Love (2002) evaluated these types using incidental unsupervised learning and found that Type IV was easier than Type II. Kurtz et al. (2013) evaluated a variety of manipulations and found that the relationship of Type II to the other types can be changed in various ways and questioned the universality of the ordering of Type II in the classic results. Taken together, these results suggest that though there are various influences on the exact ordering and differentiation of the intermediate types, the overarching pattern of Type I being easiest and Type VI being hardest and the other types being intermediate is fairly reliable.

Given the conceptual importance of the Shepard et al. (1961) types and prior research on potential differences between classification and feature inference learning, the purpose of our research was to compare classification and feature inference learning of the classic types. Our primary research question was: Do classification and feature inference learning result in different patterns of learnability across the types because of the presence of the category labels in feature inference? In this context, the label-bias hypothesis predicts that feature inference learning induces a tendency to form rules based on the category labels starting with simple unidimensional rules and progressing to more complex rules if required. This can potentially be observed in terms of advantages for feature inference over classification learning wherever a label-based rule allows performance above chance, for example, unidimensional rules for Types I and V. This is because the bias corresponds to participants trying to form a label-based rule first over other feature-based rules and in Types I and V these rules are diagnostic and semi-diagnostic, respectively. In classification tasks there is no such bias as all stimulus features are roughly comparable in nature.

## Experiment 1

**Fig. 2:**
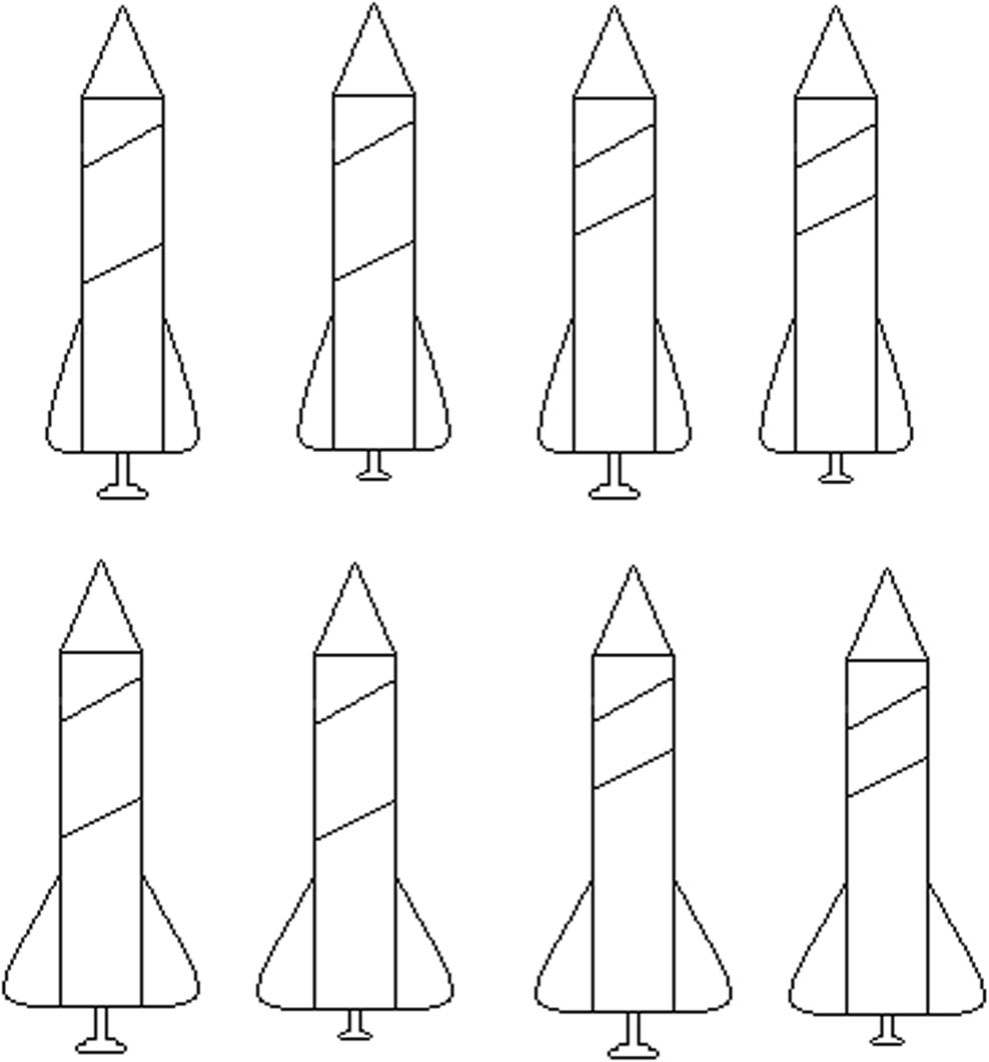
Set of eight rocket ship stimuli used in Experiment 1 composed of features from three dimensions – wing width, stripe length, and booster size

This experiment compared the learnability of a subset of the classic Shepard et al. (1961) types; specifically Types I, II, V, and VI by classification and feature inference. Not all the features in the category structure types can be unambiguously learned by feature inference. Consider Type I as shown in Table 1 for the rocket ship stimuli in Fig. 2: Suppose the participant is shown that an instance is a member of the category, “dreton,” has small wings and a small booster (A11_ in Table 1, Type I). If asked to infer what the length of the body stripe should be (A11?), there are two dreton category instances that have narrow wings and a small booster but one of them has a long stripe (A111) and the other has a short stripe (A110), so this feature inference cannot be accurately trained for this type. However, other feature inferences can be accurately learned, for example for Type I, knowing that a stimulus is a dreton with a large booster and a long stripe (A?01) only corresponds to one instance in the category structure, and thus its wing size can be unambiguously inferred as narrow (A111). In this experiment we have used Types I, II, V, and VI because these allow all feature inferences on a single dimension, dimension one, to be unambiguously trained. In contrast, Types III and IV cannot be unambiguously trained by feature inferences on any single dimension and consequently have not been evaluated here. So, all responses were on a single dimension for feature inference learning like they were in classification learning. It is also worth noting the classic finding that Types III, IV, and V are equivalent in learning difficulty.

**Table 1:** Abstract category structures for each of the two learning conditions (classification and feature inference) and the four category structure Types (I, II, V, and VI) used in Experiment 1, training phase trials at the top and testing phase trials at the bottom

Type I	Type II	Type V	Type VI
Classification Training Phase			
**A** 111	**A** 111	**A** 111	**A** 111
**A** 101	**A** 110	**A** 110	**A** 010
**A** 110	**A** 001	**A** 101	**A** 001
**A** 100	**A** 000	**A** 000	**A** 100
**B** 011	**B** 011	**B** 011	**B** 011
**B** 001	**B** 010	**B** 001	**B** 110
**B** 010	**B** 101	**B** 010	**B** 101
**B** 000	**B** 100	**B** 100	**B** 000
Feature Inference Training Phase			
A **1**11	A **1**11	A **1**11	A **1**11
A **1**01	A **1**10	A **1**10	A **0**10
A **1**10	A **0**01	A **1**01	A **0**01
A **1**00	A **0**00	A **0**00	A **1**00
B **0**11	B **0**11	B **0**11	B **0**11
B **0**01	B **0**10	B **0**01	B **1**10
B **0**10	B **1**01	B **0**10	B **1**01
B **0**00	B **1**00	B **1**00	B **0**00
Testing Phase			
**A** 111	**A** 111	**A** 111	**A** 111
**A** 101	**A** 110	**A** 110	**A** 010
**A** 110	**A** 001	**A** 101	**A** 001
**A** 100	**A** 000	**A** 000	**A** 100
**B** 011	**B** 011	**B** 011	**B** 011
**B** 001	**B** 010	**B** 001	**B** 110
**B** 010	**B** 101	**B** 010	**B** 101
**B** 000	**B** 100	**B** 100	**B** 000
A **1**11	A **1**11	A **1**11	A **1**11
A **1**01	A **1**10	A **1**10	A **0**10
A **1**10	A **0**01	A **1**01	A **0**01
A **1**00	A **0**00	A **0**00	A **1**00
B **0**11	B **0**11	B **0**11	B **0**11
B **0**01	B **0**10	B **0**01	B **1**10
B **0**10	B **1**01	B **0**10	B **1**01
B **0**00	B **1**00	B **1**00	B **0**00
A 1?1	A 1**1**1	A 1**1**0	A 1**1**1
A 1?0	A 0**0**0	A 0**0**0	A 1**0**0
B 0?1	B 0**1**0	B 0**1**0	B 1**1**0
B 0?0	B 1**0**1	B 1**0**0	B 1**0**1
A 11?	A 11?	A 10**1**	A 01**0**
A 10?	A 00?	A 00**0**	A 00**1**
B 01?	B 01?	B 00**1**	B 01**1**
B 00?	B 10?	B 10**0**	B 00**0**

*Note.* “A” and “B” refer to the two category labels and the subsequent three numbers refer to the three binary-valued feature dimensions and the feature values on those dimensions. Bolded features and question marks indicate what was queried for a given instance in a given condition. Bold features represent a correct answer and question marks indicate the lack of an unambiguous correct answer

The stimuli in this experiment, the rocket ships in Fig. 2, were used in preference to the classic color/shape/size stimuli in Shepard et al. (1961) because those stimuli don’t allow feature removability, i.e., for individual features to be removed but the stimulus to still be presented. For example, you cannot remove the shape dimension from a “large black triangle” as the coloring of the instance remains and needs to have a shape. In contrast, the rocket ship stimuli can be presented with individual features removed so that those features can be queried in terms of feature inference.

### Materials and methods

#### Participants

One hundred and twenty Cardiff University students participated for either course credit or payment. Thirty participants were trained on each of the category structure types (Table 1) with 15 in each learning condition, classification, or feature inference.

#### Materials and procedure

The abstract category structures corresponding to the four types – I, II, V, and VI – and the specification of all training and testing trials, by type, are shown in Table 1. Each category structure type has four instances in two categories, the top of Table 1. Each instance is composed of a category label, either A or B, and three binary-valued feature dimensions. Each column indicates one feature dimension with two feature values. Bold features and question marks indicate the feature that was queried on a trial. For example, the classification condition training item **A**111 indicates that the category label was queried and the feature inference training item A**1**11 indicates that the first perceptual feature dimension was queried. In the learning phase instances, bold features indicate the correct answer. The question marks on testing trials indicate that there was no unambiguous correct answer. Testing trials, at the bottom of Table 1, omitted feedback and included all classification and feature inference training items from both training conditions; a given participant was only trained on classification or feature inference but was tested on both in the testing phase. Testing also included a selection of feature inferences on the second and third dimensions as shown at the bottom of the table.

The eight rocket ship stimuli used (Fig. 2) corresponded to the eight instances in each category structure type (Table 1). The rocket ships had features on three dimensions: wing width (wide or narrow), stripe length (long or short), and booster size (large or small). The two categories of rocket ship were labelled “dreton” and “rilbar.” The assignment of physical features (Fig. 2) to abstract category features (Table 1) was randomized across participants.

Stimuli were presented using DirectRT. Participants completed 320 training trials consisting of the eight category instances in random order within a block for 40 blocks of training. Subsequent to training, participants were tested without feedback on a block of the classification training instances, followed by a block of the feature inference training instances and, finally, a block of the selection of feature inferences on the second and third dimensions. The order of the testing trials was randomized within each block.

In the training phase, after each classification training response, feedback contained the full stimulus, the words, “correct” or “incorrect” followed by “This is a dreton” or “This is a rilbar” depending on the correct answer. After each feature inference training trial there was feedback that contained the full stimulus, the words “correct” or “incorrect” followed by “The correct answer is shown above.”

Participants were given a cover story that they were visiting an alien solar system and needed to learn about the different types of rocket ships used by the aliens. They were told that they were going to be shown rocket ships and would need to make a choice between two responses. In the classification conditions, they were told that the category labels would be their response options, and in the feature inference conditions, they were told that two features would be their response options. They were made aware that they would have to guess initially but that they could learn the correct responses with practice. They then completed the learning phase followed by the testing phase.

#### Design

This was a between-subjects design with eight conditions: four structure types (Types I, II, V, and VI in Table 1) learned by either classification or feature inference. The key dependent variable was accuracy by block for each condition. We also report the proportion of participants whose performance was greater than a learning criterion as an alternative measure of learning.

#### Analysis

Analysis included standard parametric statistics as well as bootstrapped 95% confidence intervals for most results. The confidence intervals were used to relax the normality assumption.

### Results

**Fig. 3:**
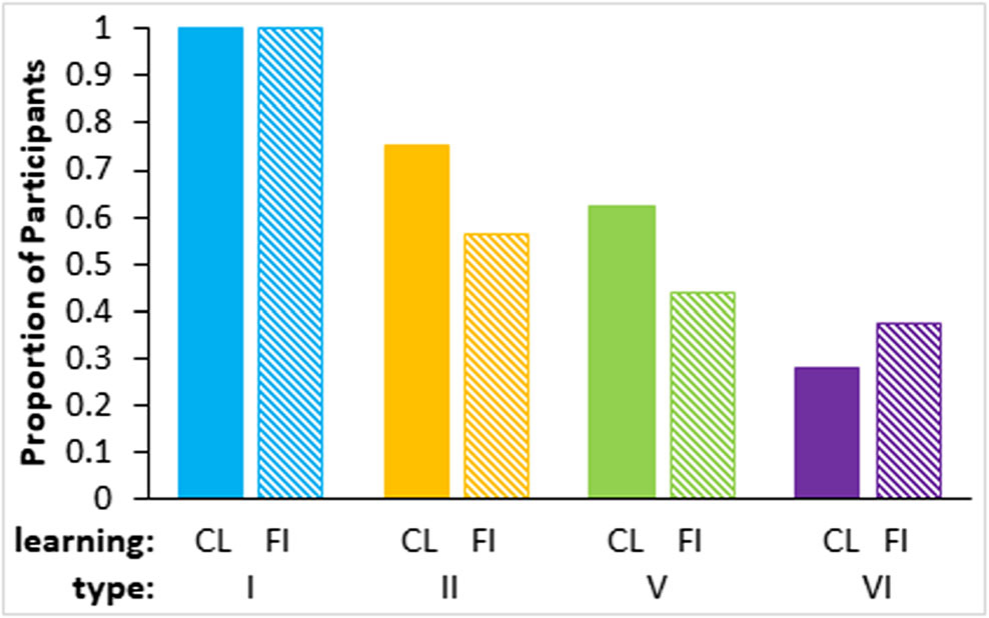
Proportion of the n = 15 participants in each learning condition and type from Experiment 1 who achieved the learning criterion (greater than 75% accuracy in the final four training blocks)

**Fig. 4:**
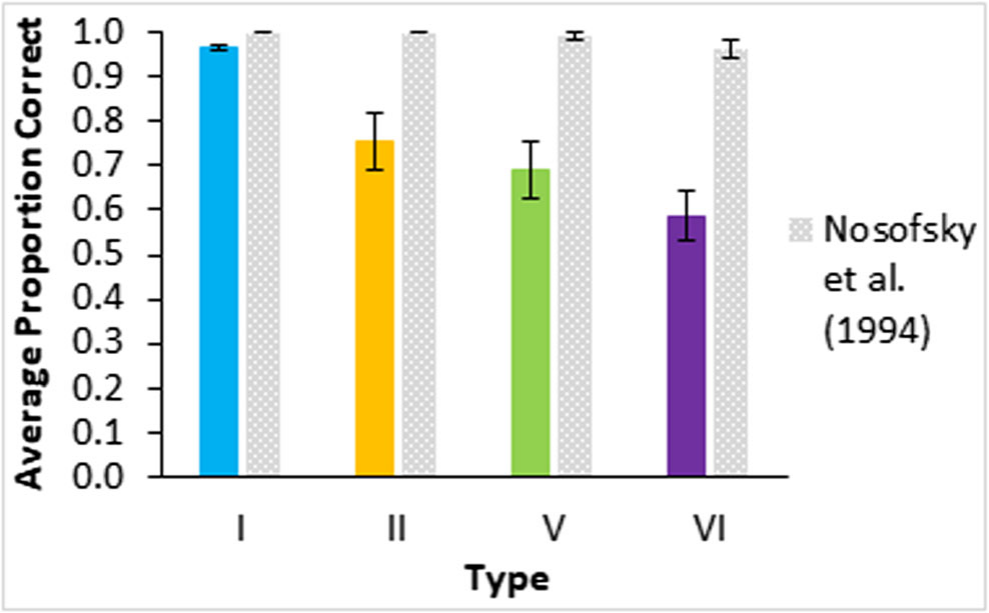
Accuracy in terms of proportion correct averaged in the final learning phase block by type for Experiment 1 classification, colored bars, and data from Nosofsky et al. (1994a), gray bars. Error bars are standard error

Asymptotic learning was fairly poor for all conditions except Type I as shown by the proportions of participants who reached a learning criterion of greater than 75% correct in the last four blocks of training (Fig. 3). Despite 40 blocks of training, only two (out of 15) participants achieved the criterion in Type VI feature inference. Learning in this experiment was substantially worse than a prior standard replication (Nosofsky et al., 1994a; see Fig. 4) that is, the average proportion correct in the last 16 trials of classification training was worse for Type II in this experiment than in Nosofsky et al. (*t*(14)=3.9, *p*=0.002, effect size as Cohen’s *d*=3.7; bootstrapping gave a 95% bias corrected confidence interval of (0.1, 0.4) and *p*=0.006). Proportion correct was also worse for Type V (*t*(14)=4.7, *p*<0.001, *d*=1.6; bootstrapped confidence interval (0.2, 0.4) and *p*=0.001) and for Type VI (*t*(18)=6.2, *p*<0.001, *d*=2.9; bootstrapped confidence interval (0.3, 0.5) and *p*<0.001). Despite this there is evidence that some learning occurred in all conditions as performance is significantly above chance for Type II (*t*(14)=4.0, *p*=0.001, *d*=0.996; bootstrapped confidence interval (0.1, 0.4) and *p*=0.005) and Type V (*t*(14)=3.0, *p*=0.009, *d*=1.10; bootstrapped confidence interval (0.1, 0.3)) and there is a marginal difference in the right direction for Type VI (*t*(14)=1.5, *p*=0.14, *d*=0.065; bootstrapped confidence interval (-0.02, 0.2)). Note that the degrees of freedom for some of the prior and subsequent t-tests are adjusted degrees of freedom in the context of assuming unequal variances.

**Fig. 5:**
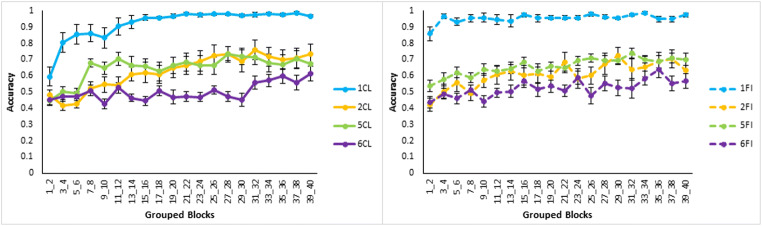
Averaged accuracy in terms of proportion correct across groups of two training blocks by Type (I, II, V, and VI) and learning condition (CL = Classification, FI = Feature Inference) in Experiment 1. Classification learning is displayed on the left, and feature inference learning on the right. Error bars are standard error

For the classification learning task (see Fig. 5, left panel) average accuracy over all learning blocks was higher for Type I than the next most accurate type, Type V (*t*(19)=5.5, *p*<0.001, *d*=2.02; bootstrapped confidence interval (0.2, 0.3) and *p*=0.001). There was no significant difference between Types V and II (*t*(28)=0.7, *p*=0.50, *d*=0.25; bootstrapped confidence interval (-0.2, 0.1) and *p*=0.50), but average accuracy was significantly higher for Type II than Type VI (*t*(15)=2.7, *p*=0.016, *d*=0.996; bootstrapped confidence interval (0.04, 0.2) and *p*=0.022). Overall, the results of classification learning replicate the classic difficulty ordering, though Types II and V were not clearly differentiated.

For the feature inference learning task (Fig. 5, right panel), average accuracy was significantly higher for Type I than Type V (*t*(21)=12.0, *p*<0.001, *d*=4.39; bootstrapped confidence interval (0.3, 0.4) and *p*<0.001). Type V was not significantly different from Type II (*t*(20)=1.0, *p*=0.34, *d*=0.36; bootstrapped confidence interval (-0.1, 0.1) and *p*=0.34) nor was Type II significantly different from Type VI (*t*(21)=1.5, *p*=0.14, *d*=0.56; bootstrapped confidence interval (-0.02, 0.2) and *p*=0.15). Thus, feature inference learning only clearly replicated the classic finding in terms of Type I being the easiest with poor differentiation of Types II, V, and VI.

Direct comparison of classification and feature inference learning by type shows that there were significantly higher accuracies for Type I feature inference than for Type I classification in the first two blocks of learning (*t*(25)=3.4, *p*=0.002, *d*=1.24; bootstrapped confidence interval (0.1, 0.4) and *p*=0.007). This superior performance in feature inference supports the label-bias hypothesis that feature inference learning induces a bias to evaluate the label-based unidimensional rule and classification does not as the label was not present. The average accuracies for classification and feature inference learning across all learning blocks were not significantly different for Type II (*t*(28)=0.3, *p*=0.74, *d*=0.12; bootstrapped confidence interval (-0.1, 0.1) and *p*=0.74), for Type V (*t*(21)=0.3, *p*=0.80, *d*=0.096; bootstrapped confidence interval (-0.1, 0.1) and *p*=0.79) or for Type VI (*t*(28)=0.9, *p*=0.36, *d*=0.33; bootstrapped confidence interval (-0.1, 0.02) and *p*=0.37).

**Fig. 6:**
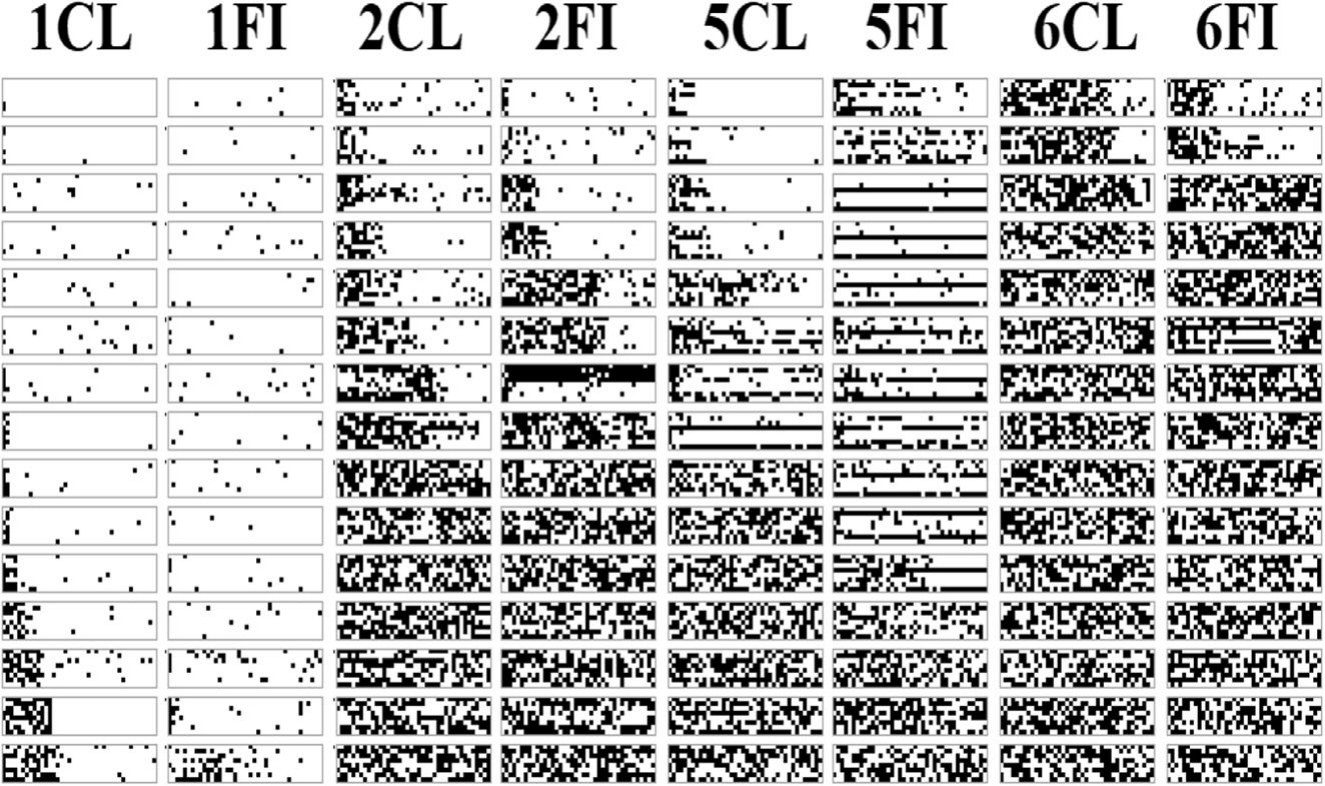
Panels showing the individual performance of each participant in Experiment 1 on every learning trial. White dots indicate a correct answer on an individual trial and black dots indicate an incorrect answer. Each row represents a single category instance, and the instances are ordered as in Table 1. Therefore, each row within a panel shows performance on one specific trial across the 40 learning blocks. Each column of panels represents a learning condition as indicated by the column headers

As a way of visualizing learning at the level of individual participants, the learning error diagrams in Fig. 6 show responding on each learning trial for every participant arranged by learning type and condition: Each participant’s responding is shown within a gray rectangle outline, within this outline a trial is a single dot, and black dots indicate response errors. Each column of dots is the response accuracy for each training item in a block in standardized order, as in Table 1, and each row of dots in a rectangle represents the accuracy for a given training item across all 40 training blocks. Finally, participants in each condition have been arranged roughly by their learning performance, good learners toward the top and poor learners toward the bottom.

A key benefit of error diagrams is to be able to spot patterns in errors at the level of individual participants. Amongst these, perseverative suboptimal rule use can be seen (Fig. 6) as systematic errors on particular instances, i.e., as horizonal black lines. Such suboptimal rule use is most apparent for Type V feature inference where a label-based rule allowed 75% accuracy at the cost of consistent errors on the fourth and eighth instances in Table 1. Participants were operationally defined as using this suboptimal rule if their responding was consistent with this error pattern for over 15 blocks out of 40 (with a maximum allowed deviation from the pattern of one response per block). Approximately half of the participants in the Type V feature inference learning condition showed this pattern of responding, significantly more than in classification learning (*p* = 0.018, Fisher’s exact test). Crucially, this occurred despite the existence of a corresponding suboptimal rule based on a single feature dimension also being available in classification learning. This suggests perseveration with a label-based rule that supports the label-bias hypothesis.

Finally, the error diagrams show rapid transitions from chance performance to high accuracy, seen as a change from the left (noise) to the right (white) of an individual panel. These rapid transitions are consistent with rule acquisition as finding a rule that gives optimal performance allows for rapid performance improvement.

### Discussion

The results of this experiment support the label-induced rule-bias hypothesis that the category labels in feature inference learning bias participants to try to form label-based rules. This hypothesis is supported by a feature inference learning advantage for Type I, the presence of significantly more sub-optimal rule use for Type V feature inference compared to Type V classification and the similarity in the learning curves for Types II, V, and VI feature inference.

In more detail, the label-bias for Type I manifests as follows: In classification learning three unidimensional rules are all roughly equivalent, one for each feature dimension, with no clear basis for an initial preference between them, whereas inference has a single label-based unidimensional rule that is distinct from the other feature-based rules. We argue that these differences arise out of a tendency to start with the label-based rule in feature inference, and therefore participants achieved perfect performance more rapidly in Type I. For Type I classification, learning occurred more slowly due to the lack of a clear basis for a preference between the three unidimensional rules. Some participants took longer than others, and this greater variability resulted in classification participants, on average, taking longer to find the correct rule; they achieved perfect accuracy more slowly.

Further support for the label-bias hypothesis comes from the perseveration of suboptimal rule use in Type V feature inference. A possible reason for this perseveration is in terms of difficulty as the relatively poor performance on the task overall compared to Nosofsky et al. (1994a) and the interaction of this with the difference between classification and feature inference: The suboptimal label-based rule is easier to find in feature inference due to the bias and gives accurate enough performance to encourage participants to keep using the rule given the task difficulty.

Finally, the greater similarity of the learning curves for Types II, V, and VI feature inference (Fig. 5) supports the label-bias as the bias is consistent with attempts to form label-based rules in feature inference conditions in contrast to classification. In Type I this leads to accurate performance early on in learning as is observed as the label-based rule is accurate. However, a bias for simple unidimensional rules for the harder types, does not allow optimal performance. In addition, even with the label-bias, there are still multiple nonoptimal rules for the higher types involving the labels. So, a label-bias for the higher types is less helpful for performance and may actually be harmful, manifesting in similar, relatively poor learning across the types.

The label-bias hypothesis could be taken to imply that feature inference induces rule representation and that classification does not. However, the error diagrams suggest rapid transitions from chance performance to near perfect performance consistent with the use of rules in both classification and feature inference learning tasks for people who learned. The key difference is in terms of the bias on rule formation in feature inference rather than a wholly different class of representation such as exemplars.

Perhaps the most surprising aspect of these results is the poorer learning of the classification conditions compared to Nosofsky et al. (1994a): It is clear some learning occurred in all conditions, just not as much (see Fig. 4). Methodologically, the classification learning conditions here were similar to standard replications with the key exception of the stimuli.

The current rocket ship stimuli are not unusual for the perceptual categorization paradigm where many prior studies have used rocket ships (see Hoffman & Ziessler, 1983; Johansen et al., 2015; Palmeri, 1999; etc.). Also, different features were not visually hard to discriminate (see Fig. 2). Kurtz et al. (2013) argued that the verbal nameability of the feature values, the ease with which a feature can be given a verbal descriptor, influenced learnability; the nameability of Types II and IV impacts how well they are learned and can reverse the typical ordering for these types. The implication of nameability is in terms of the implied interaction with the formation of verbal rules and their memorability. Minda, Desroches, and Church (2008) found that when a naming task is given to children prior to learning Type II, this improves performance. To improve the learning in the classification conditions to be more consistent with prior replications, we adjusted the verbalizability of the stimuli in Experiment 2.

## Experiment 2

To improve the nameability of the features on each dimension and reduce interference/confusability between dimensions, Experiment 2 changed the dimensions so that they were color, shape, and size, as in the Shepard et al. (1961) stimuli, but applied to the rocket ship features, and the category labels were changed from two syllables to one syllable. In combination, these changes were intended to facilitate learning via more compact verbal rules. Importantly, rule use was assessed by asking participants to report what they saw and how they responded using qualitative questions at the end of the experiment. Finally, the feature inference feedback was amended slightly to include the category label, and the testing phase for both learning tasks was updated to include all possible feature inferences.

### Materials and methods

#### Participants

Four hundred and sixteen Cardiff University students participated for either course credit or payment. Each of the eight combinations of type (Types I, II, V, and VI) and learning condition (classification or feature inference) had 52 participants.

#### Materials and procedure

**Fig. 7:**
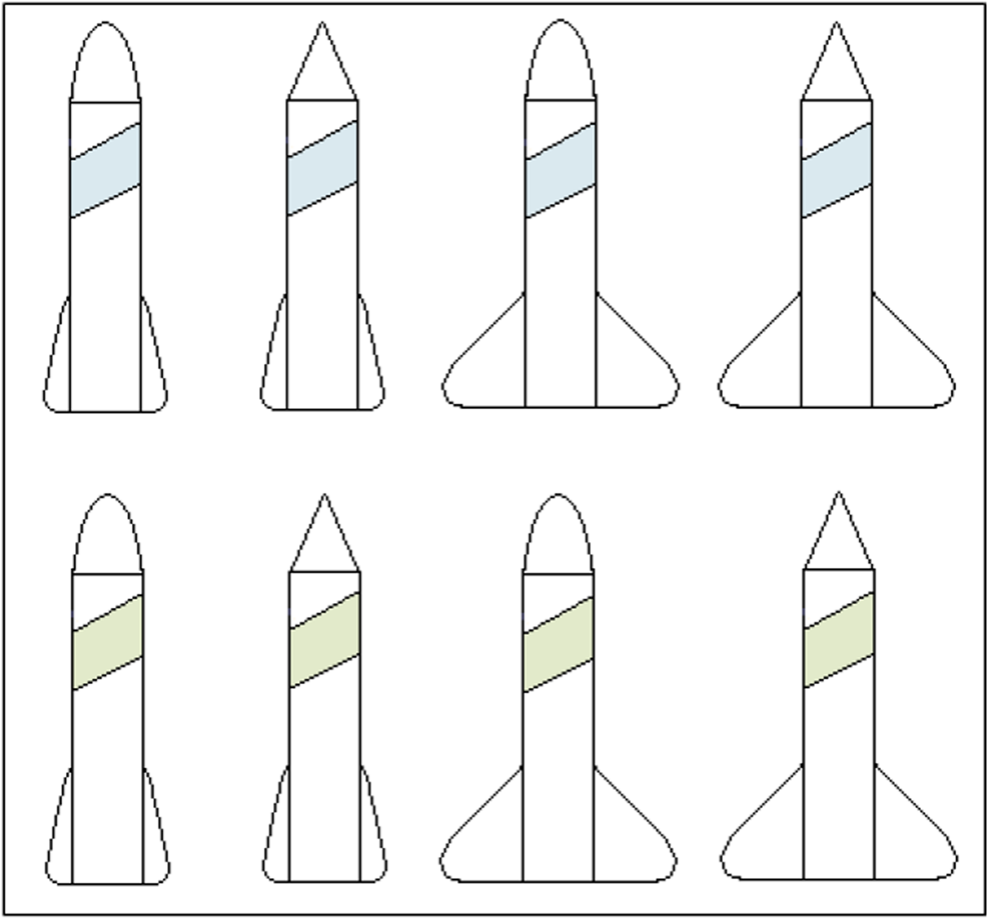
Rocket ship stimuli used in Experiment 2, composed of features on three stimulus dimensions: blue/green stripe, pointed/rounded cone, and wide/narrow wings

The eight new rocket ship stimuli were composed of three feature dimensions: stripe color (blue or green), cone shape (pointed or rounded), and wings size (wide or narrow) as shown in Fig. 7. The category labels were changed to “thab” and “lork”. The feature inference feedback was adjusted to include the same information as the classification feedback such that it included the label presented under the stimuli as well as the feedback, “correct” or “incorrect” and a note that the correct answer was shown at the top of the screen. The testing phase included all the original classification and feature inference items in Experiment 1, but feature inference trials were added to include all possible feature inferences on the training items (see Appendix 1). Also, at the end of the experiment, participants were given the following questions: “Did you find a rule to help you learn the task? If so, please describe it briefly.” Followed by: “If you did not learn a rule what did you use/learn to help you do the task?” All other methodological details of this experiment were the same as Experiment 1.

### Results

**Fig. 8:**
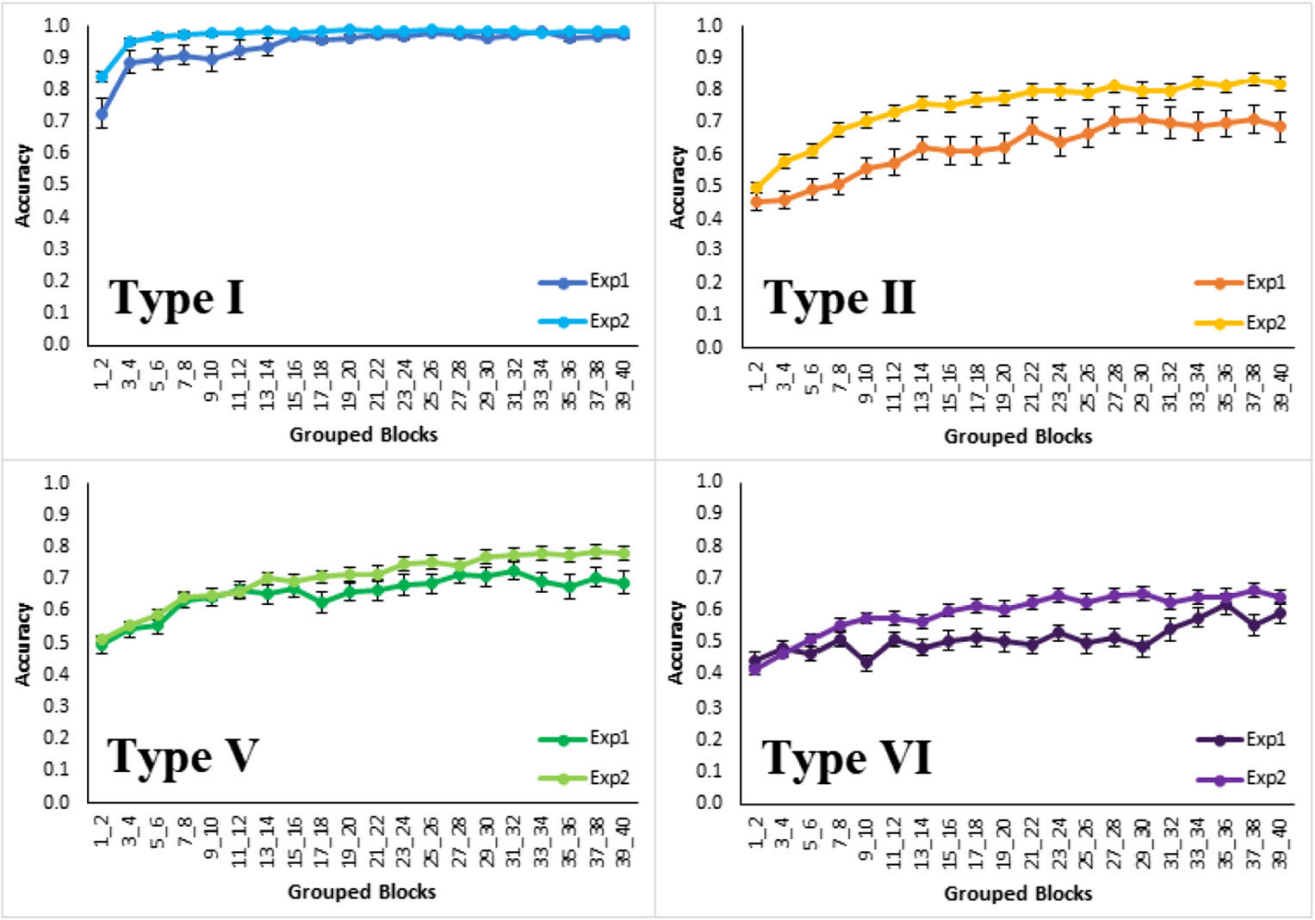
Comparison of accuracy as average proportion correct by type across groups of two training blocks between Experiments 1, dark lines, and 2, light lines. Error bars are standard error

The updated stimuli improved learning in all of the types (see Fig. 8) with significant differences for Type I (*t*(33)=3.0, *p*=0.006, *d*=0.62; bootstrapped confidence interval (0.01, 0.06) and *p*=0.019), Type II (*t*(132)=3.4, *p*=0.001, *d*=0.71; bootstrapped confidence interval (0.1, 0.2) and *p*=0.001) and Type VI (*t*(117)=4.1, *p*<0.001, *d*=0.85; bootstrapped confidence interval (0.04, 0.1) and *p*<0.001), and a marginally significant improvement in Type V (*t*(61)=1.8, *p*=0.08, *d*=0.37; bootstrapped confidence interval (-0.1, 0.005) and *p*=0.08. Note, some degrees of freedom are adjusted from assuming unequal variances). Overall, these results are consistent with more compact and less confusable verbal rules facilitating learning by being somewhat easier to use.

**Fig. 9:**
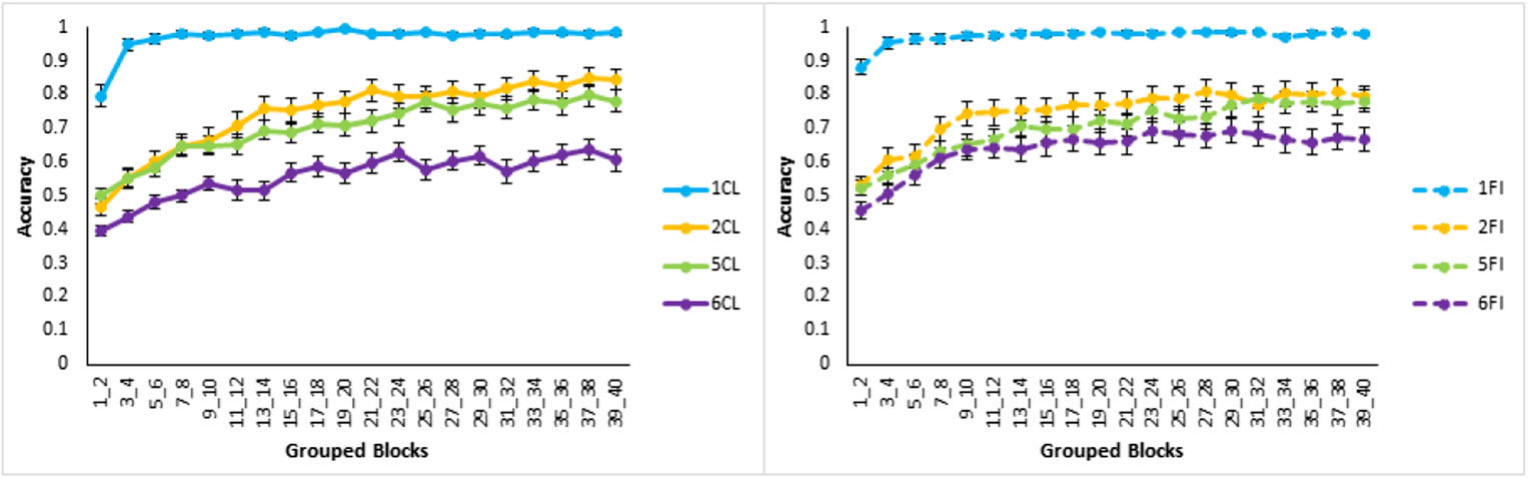
Accuracy averaged in terms of proportion correct by type and learning condition with classification learning on the left, and feature inference learning on the right for Experiment 2. Error bars are standard error

For average accuracy across all learning blocks in classification learning (Fig. 9 left side) accuracy was significantly higher for Type I than the next closest type, Type II (*t*(54)=9.1, *p*<0.001, *d*=1.79; bootstrapped confidence interval (0.2, 0.3) and *p*<0.001). There was no significant difference between Types II and V (*t*(102)=1.3, *p*=0.21, *d*=0.26; bootstrapped confidence interval (-0.02, 0.1) and *p*=0.21) and Type V was significantly higher than Type VI (*t*(102)=5.0, *p*<0.001, *d*=0.98; bootstrapped confidence interval (0.09, 0.2) and *p*<0.001). Thus, the results of classification learning replicate the classic difficulty ordering, though Types II and V were again not clearly differentiated.

For average accuracy across all learning blocks in feature inference (Fig. 9 right side), Type I was significantly higher than Type II (*t*(53)=8.2, *p*<0.001, *d*=1.61; bootstrapped confidence interval (0.2, 0.3) and *p*<0.001) and there was no significant difference between Types II and V (*t*(99)=1.2, *p*=0.22, *d*=0.24; bootstrapped confidence interval (-0.03, 0.1) and *p*=0.23) but there was a marginally significant difference between Types V and VI (*t*(102)=1.8, *p*=0.07, *d*=0.35; bootstrapped confidence interval (-0.005, 0.1) and *p*=0.07). Thus, feature inference learning replicated the general ordering in Experiment 1 with Type I being the easiest.

Direct comparison of classification and feature inference learning by type shows that Type I feature inference had higher accuracy than classification across the first two learning blocks (*t*(88)=2.8, *p*=0.007, *d*=0.55; bootstrapped confidence interval (0.03, 0.2) and *p*=0.008). This replicates Experiment 1 and supports the label-bias hypothesis. It is worth noting that a significant difference occurred despite the improvement in performance from the updated stimuli raising performance towards ceiling. Further, Type II feature inference had higher accuracy than classification across the first two learning blocks (t(92)=2.2, p=0.028, *d*= 0.43, bootstrapped confidence interval (0.007, 0.1)). Additionally, feature inference had higher accuracy than classification for Type VI when averaged across all blocks of learning (*t*(96)=2.6, *p*=0.012, *d*=0.51; bootstrapped confidence interval (0.02, 0.1) and *p*=0.012). Finally, there was no significant difference in accuracy across all learning blocks in the Type V conditions (*t*(102)=0.02, *p*=0.98, *d*=0.004; bootstrapped confidence interval (-0.06, 0.06) and *p*=0.98). So, Types I, II, and VI indicate differences between classification and feature inference learning and support the label-bias hypothesis, but Type V does not, possibly due to easier stimuli reducing the use of suboptimal dimensional rules.

The difference between classification and feature inference (Fig. 9) is further supported by the marginally significant interaction between learning conditions and types for Types V and VI (*F*(1,204)=3.38, *p*=0.067, *hp2*= 0.016). This interaction is consistent with a difference between classification and feature inference in terms of poorer differentiation of the learning conditions in feature inference than in classification. However, the predominant reason for the poorer differentiation in feature inference is the significantly better performance for Type VI by feature inference than classification learning.

**Fig. 10:**
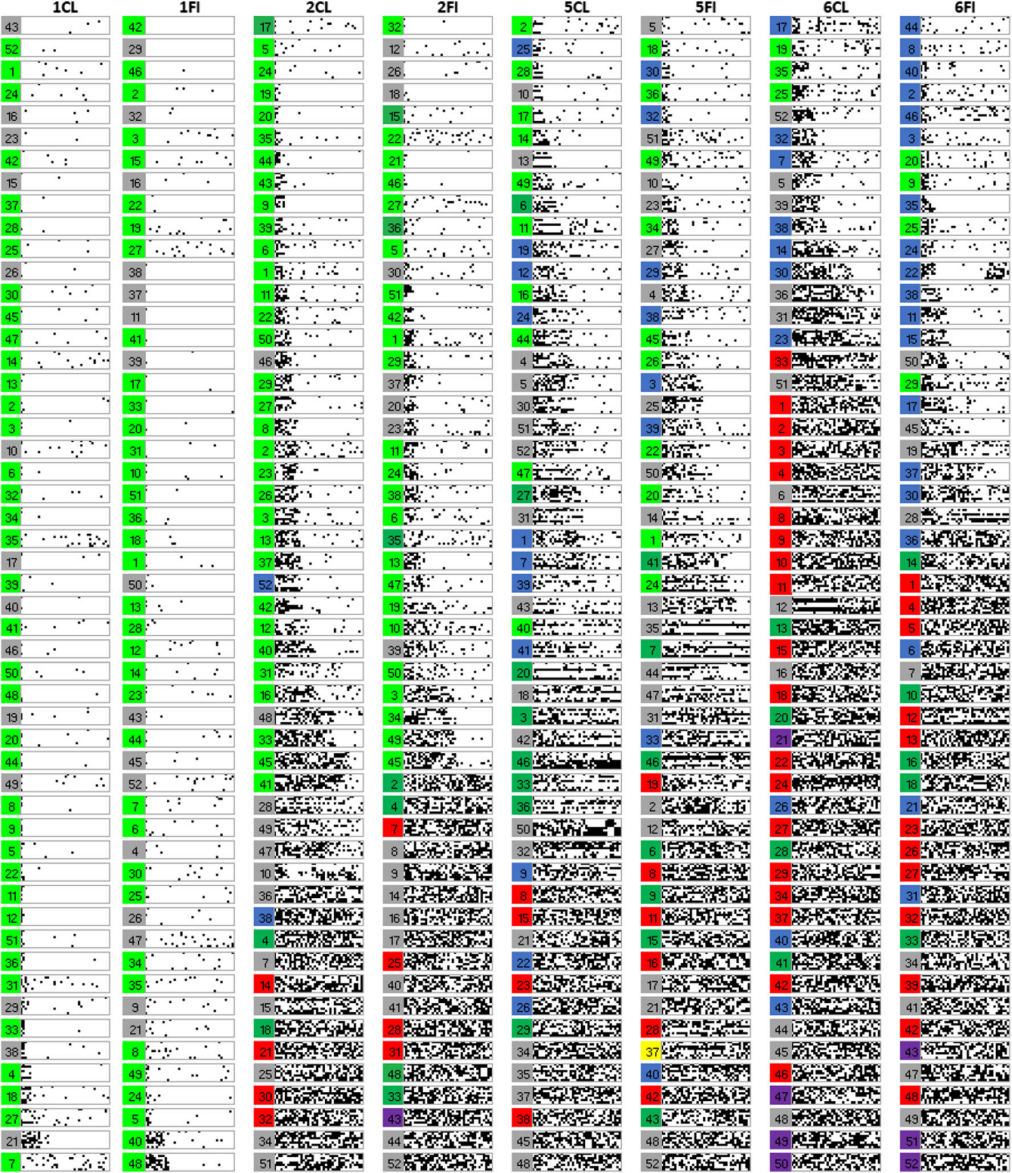
Panels show individual performance of each participant in Experiment 2 on every learning trial. White dots indicate a correct answer on an individual trial and black dots indicate an incorrect answer. Each row represents a single category instance, and the instances are ordered as in Table 1. Therefore, each row within a panel shows performance on one specific trial across the 40 learning blocks. Each column of panels represents a learning condition as indicated by the column headers. Blocks of color to the left of each panel represent the learning strategy as inferred from the questions at the end of the experiment (light green = optimal rule, dark green = suboptimal rule, red = no rule, blue = exemplars, gray = ambiguous, yellow = prototypes, purple = pattern of responding)

As in Experiment 1, individual participant error diagrams (Fig. 10) show rapid transitions from chance performance to high accuracy (formalized in detail below), consistent with the sudden acquisition of a rule. There was far less use of suboptimal rules, especially for Type V relative to the previous experiment, as might be expected from better learning due to the updated stimuli. As supported by responses to the questions about learning strategy (discussed below), this is consistent with more participants finding optimal rules.

At the end of the experiment, participants described how they learned the task. These qualitative data were used to assign participants to seven groups in terms of those who used the optimal rules, suboptimal rules, “no rule”/poor reported learning, prototypes, a pattern of responding, memorized exemplars, or whose descriptions were ambiguous. The criteria for coding into these groups is described in Appendix 2.

**Fig. 11:**
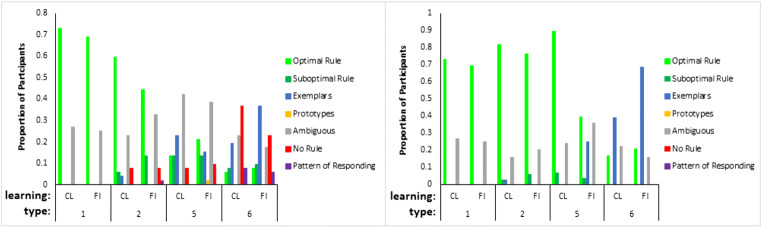
Proportion of participants who reported using each kind of representation for each type and learning condition. The left panel includes all participants and the right panel is for participants who met the learning criterion (where the number of participants who learned in each type and condition can be seen in the error diagrams; Fig. 10)

The proportion of the participants attributed to each strategy for each learning condition (Fig. 11, left panel) shows a predominance of optimal rule use, 47% (participants marked by light green patches, Fig. 10) across Types I, II, and V for participants in the error diagrams, compared to the next most prevalent strategy. As seen in the error diagrams for Type VI, the majority of participants did not learn the task. For all types, participants who reported that they did not learn, the red patches in Fig. 10, were mostly participants in the error diagrams who clearly did not learn anything. When the participants who did not learn were removed (Fig. 11, right panel), the proportion of optimal rule users averaged across all types was even higher, 64%. Despite the complexity of the Odd-Even rule in Type VI, 21% of the participants who learned the task made a statement that was clearly consistent with using this rule. Although there were more participants who reported exemplar memorization (the blue patches in Fig. 10) for the harder types, inclusion in this group was based on the lax requirement of participants’ stating that they were using exemplars rather than a requirement to list all exemplars. In contrast, the attribution to the rule use group was based on the harsh requirement not only to specify a rule but a confirmation that their rule was accurate given the stimuli they saw. Overall, the accuracy/believability of participants statements about their performance and strategy was objectively very high; when they said they didn’t learn, they hadn’t learned. When they reported an optimal rule, they had learned, but when their descriptions were ambiguous or reported memorizing exemplars, they may or may not have learned, and this was more prevalent for harder types. But Type II had a preponderance of participants who both reported accurate rules and learned well.

**Fig. 12:**
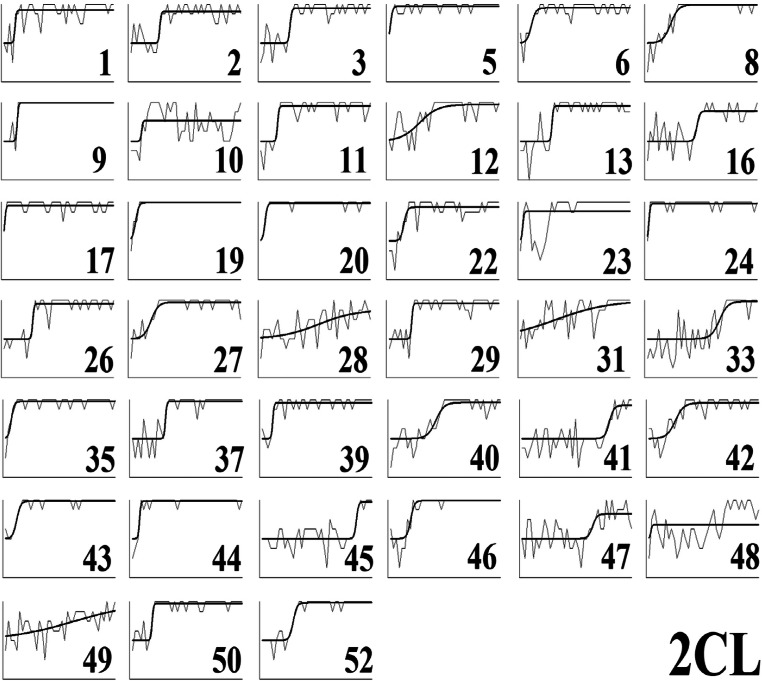
Sigmoid functions fitted to learning curves for participants who showed any learning (in the error diagrams; Fig. 10) in the Type II classification condition

To quantify the apparent sudden improvements in performance observed in the error diagrams (Fig. 10), we used Boltzmann sigmoid functions fitted using Origin (Pro) Version 2017 to individual participant learning curves to infer the interval between initial and final learning performance (see Appendix 3 for details). To emphasize, this was not directly fitting a category representation model, for example, ALCOVE (Kruschke, 1992), to participants’ data and using the goodness of those fits to infer the nature of the representation, for example, exemplar representation. Rather the slope (the steepness) of the threshold for the fitted sigmoid functions, for example, as in the individual participant learning curves in Fig. 12 for Type II classification learning, indicates how suddenly participants improved from initial chance performance to their final level of accuracy; specifically, this is formalized in terms of the number of learning blocks between the start and end of performance improvement, i.e. the block just before where performance clearly started to improve and the block later in learning where it stopped improving. Short acquisition intervals, we argue, are evidence of sudden rule insight whereas long acquisition intervals are indicative of gradual build-up in associative strengths between categories and a representation such as exemplars.

The fitted sigmoid functions to the learning curves for each individual participant in Type II classification (see Fig. 12 and Appendix 3) show that these functions do a good job of characterizing the rapidness of performance improvement. Most participants in this condition showed extremely rapid performance transitions consistent with sudden rule acquisition, for example, participant 2, but a small number of participants learned gradually, consistent with exemplar memorization, for example, participant 28.

**Fig. 13:**
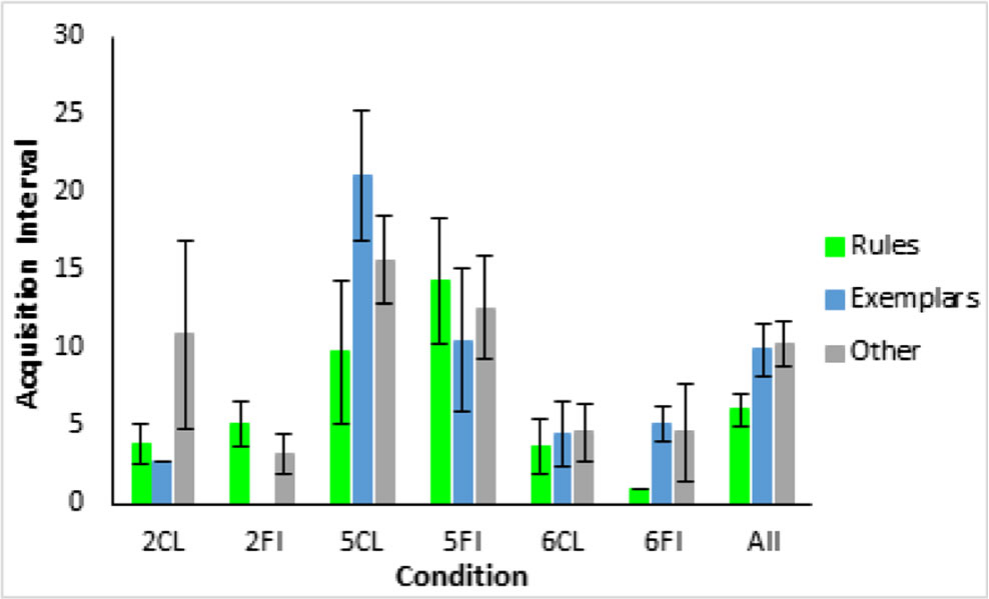
Average speed of acquisition for participants in Experiment 2, split by their qualitative grouping (optimal rules = green, exemplars = blue, other = gray). Error bars are standard error

**Fig. 14:**
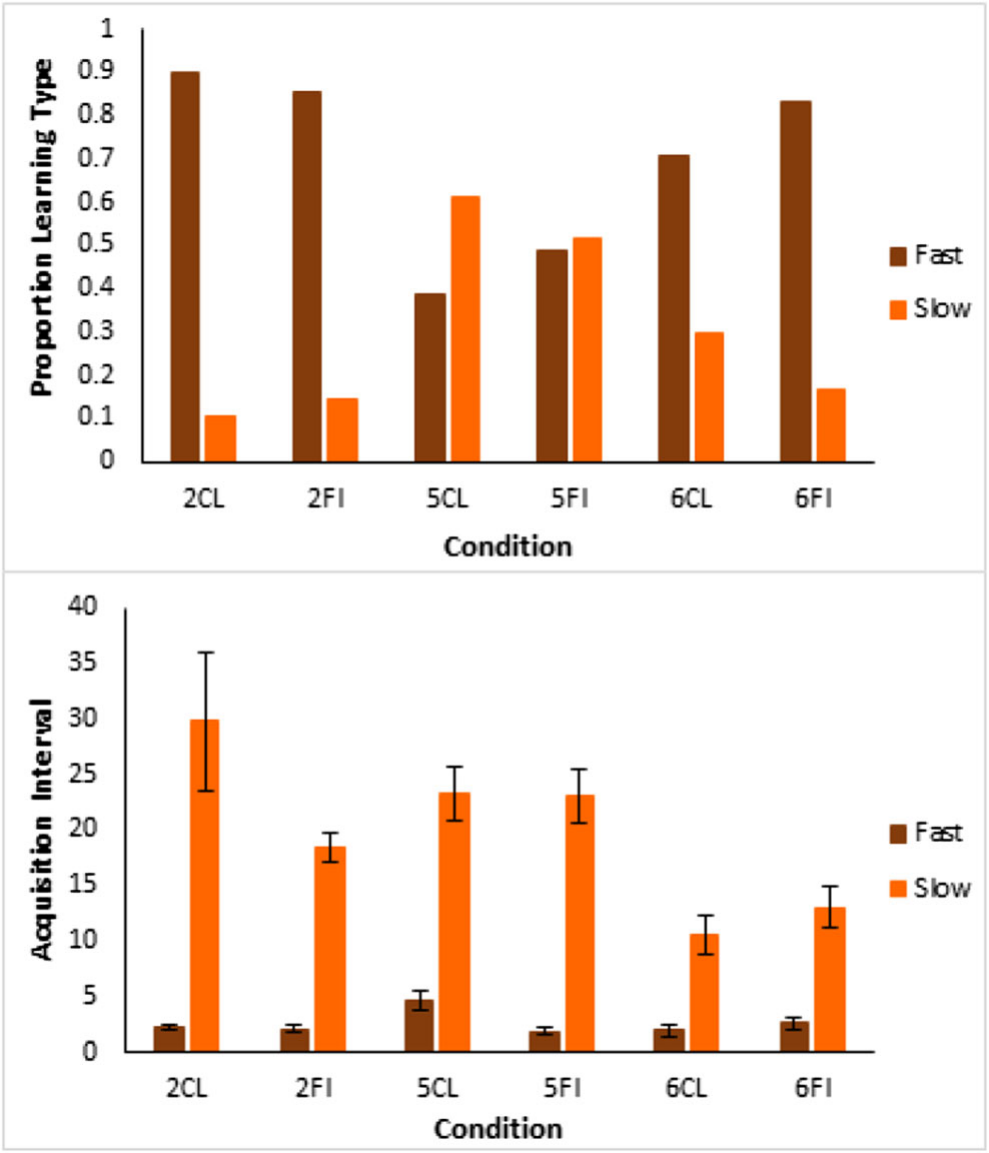
Proportions of participants who showed rapid versus slow improvements in task performance by condition in Experiment 2 (**top panel**), and average acquisition intervals for fast versus slow participants by condition (**bottom panel**). Error bars are standard error

Average speed of acquisition across conditions by qualitative groupings (Fig. 13) shows that participants who reported using (optimal) rules learned at least marginally faster on average over all conditions than those who reported memorizing exemplars (*t*(117)=2.0, *p*=0.043, *d*=0.39; bootstrapped confidence interval (0.1, 7.9) and *p*=0.058) or other strategies (*t*(119)=2.3, *p*=0.02, *d*=0.38; bootstrapped confidence interval (0.8, 7.8) and *p*=0.02). Splitting participants using their sigmoid slopes into slow learners, slope <= 0.05, and fast learners, slope > 0.05, shows that only a small proportion, 10% in the top panel of Fig. 14, of people who learned at all learned slowly in Type II classification, and that the elimination of this handful of slow participants left participants with a very short average acquisition interval of 2.2 blocks (Fig. 14, bottom panel), consistent with rapid rule acquisition. The Type II feature inference results (see Appendix 4) show a similar pattern supporting rapid rule acquisition, and both are consistent with the preponderance of reported rule use in Type II. For Types V and VI the proportions of participants who learned slowly by this criterion were substantially larger (Fig. 14, top panel), consistent with the greater reported use of exemplar memorization in the qualitative results (Figs. 11 and 10). It is worth noting that Type V was learned even more slowly than Type VI, possibly because the use of a verbal rule in Type V still requires the memorization of at least two exception instances to achieve perfect performance.

**Fig. 15:**
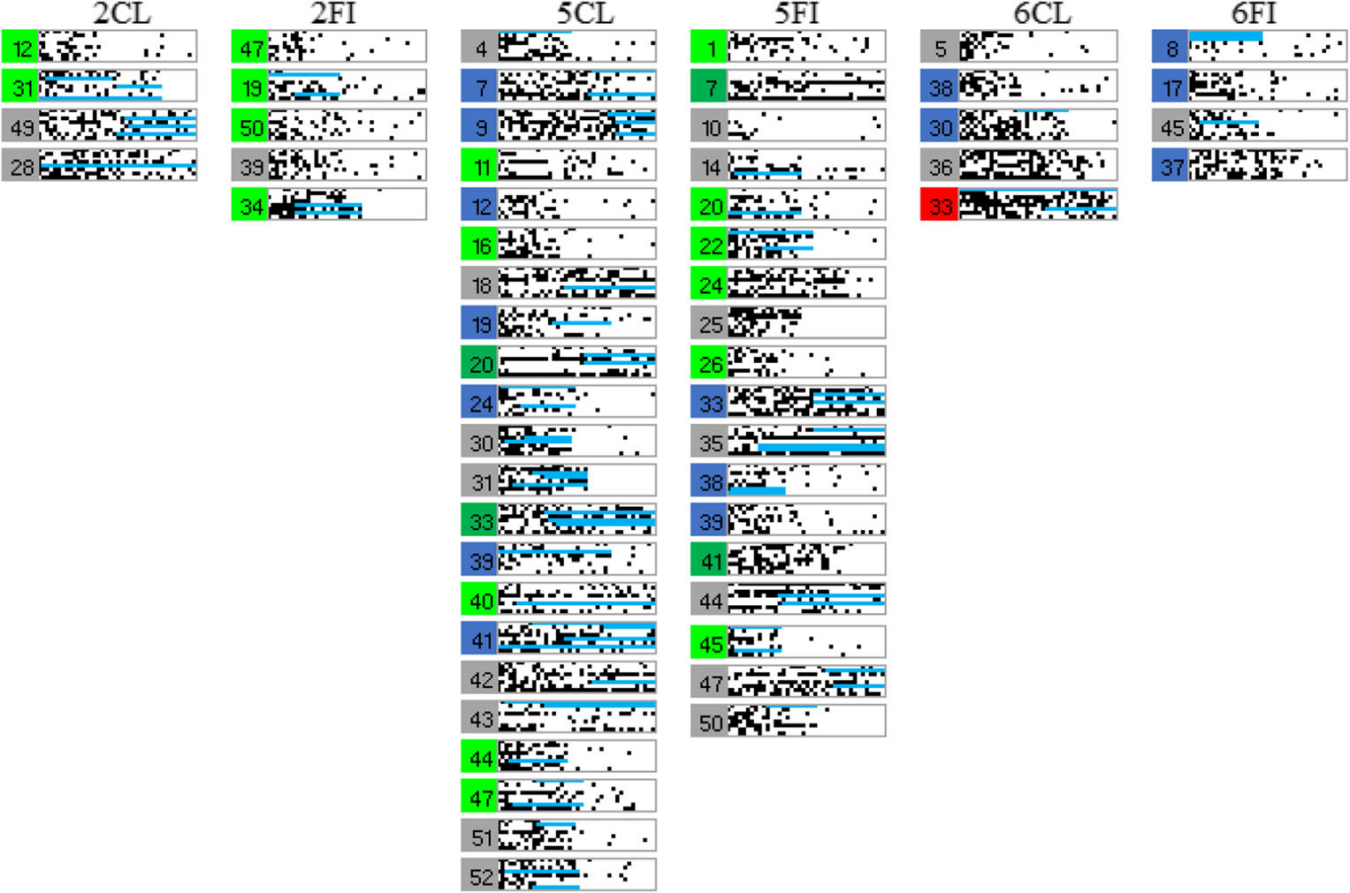
Error diagrams for participants who learned slowly (sigmoid slope <=0.05) for Types II, V, and VI. The light blue bars correspond to sequences of at least 13 blocks in a row correct for specific category instances in the presence of clearly poor performance on at least one other instance, i.e., performance at chance

As a compliment to the speed of acquisition analysis supporting rule use, the individual error diagrams specifically for slow learners (slope <= 0.05 in the last paragraph) show evidence of exemplar memorization (Fig. 15). In these error diagrams, evidence of exemplar memorization can be seen as near perfect learning on a subset of instances but with chance performance on other instances. Visually this appears in the error diagrams in Fig. 10 as single long white lines corresponding to good performance and intermixed black and white dotted lines corresponding to chance performance. The light blue bars in Fig. 15 correspond to long sequences of correct answers (at least 13) on some specific category instances in the presence of chance performance on at least one other instance (13 in a row correct is very unlikely by chance). Of the 58 slow learners (Fig. 15), 38 show this pattern clearly. However, it is worth emphasizing this is a conservative conclusion in that many of the other individuals show the pattern more weakly, and a requirement for 13 in a row correct is a strict criterion. Overall, slow acquisition seems to have arisen out of having learned some instances and not others rather than either gradual or sudden acquisition of all instances.

**Fig. 16:**
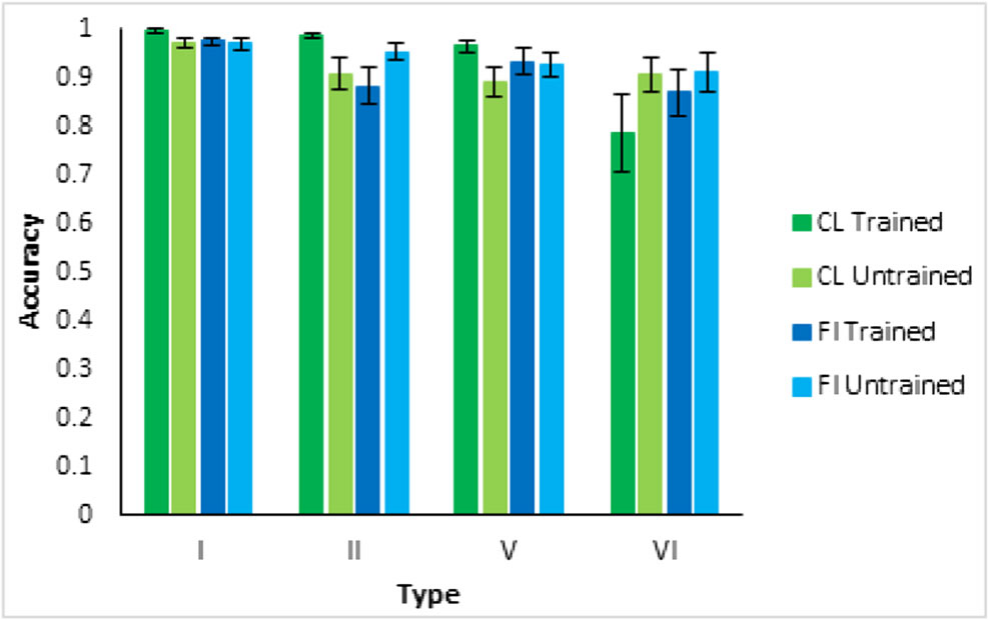
Average accuracy as proportion correct in the testing phase for each type, where the results are separated by the nature of the testing trials (classification or feature inference) and by whether or not the participants were trained on trials of that kind, all for participants who met a greater than 75% learning criterion over the last four blocks. Error bars are standard error

Participants in both learning conditions were tested on one block of classification items matching the classification training condition items and one block of feature inference items matching the feature inference training condition items, even though they were only trained on one of these learning tasks. For participants who met a greater than 75% learning criterion in the last four learning blocks, the testing trial results (Fig. 16) showed that decrements in performance between trained and untrained items were very small. For example, the largest decrement was only 0.13 in Type VI Classification. This equates to one participant out of two making slightly more than one mistake across the eight untrained testing items on average and shows very little decrement for the untrained trials, consistent with verbal rule use.

### Discussion

Observed differences between classification and feature inference learning are consistent with the label-bias hypothesis; this hypothesis was most directly supported by the Type I advantage, better performance for Type I feature inference over Type I classification early in training consistent with the bias inducing participants to try the correct rule more quickly. The hypothesis was also supported by better early learning of Type II in feature inference learning than in classification. More subtly the bias is supported by the poor differentiation between Types II, V, and VI in feature inference learning which is consistent with similar attempts to use label-based rules in all feature inference tasks even when these were not optimal rules.

Despite the support for the label-bias hypothesis in terms of differences between the learning tasks, the even stronger conclusion from these results is the substantial evidence for the use of rules in both learning tasks. This is supported by the rapid performance transitions in the error diagrams, the high accuracy on the untrained responses, and the qualitative descriptions of rules that are accurate.

In more detail, the testing trial data showed only small decrements between performance on trained and untrained classification and feature inference trials. For example, a unidimensional rule in feature inference training might have been responding with wide wings for a thab and this would make it easy to do the untrained classification item that involved responding thab when shown a rocket ship with wide wings. The verbal rule contains all elements of the stimuli and the category label, so it does not matter which response is queried.

For the qualitative data what people said they did was quite closely related to their actual performance. Bearing in mind that the strategy reported by 29% of all participants was ambiguous and only a further 6% reported a strategy that was inconsistent with their performance, the strategy reported by 65% of participants was consistent with their learning performance either in terms of saying they learned the task or saying that they didn’t learn. For example, a participant in the Type VI classification condition who did not learn the task said, “No, didn’t find a rule so I guessed each time.” Another participant in the Type VI classification condition who did learn stated, “If only one feature had changed, the top was the opposite to the previous rocket. If two changed, it was the same top as the previous rocket. If three changed, it was again the opposite.” This is the Odd-Even rule. As it seems strange that participants could verbalize accurate rules if they were not using them, these data indicate the dominance of verbal rules across all conditions. The harder conditions did show a greater proportion of qualitative results supporting exemplar memorization, but the accuracy of this characterization is less clear than for rules because participants were not asked to explicitly report all instances.

Lastly, the learning improved significantly from Experiment 1 to Experiment 2, arguably due to improvement of the nameability of the feature dimensions and values used for the stimuli and the subsequent impact on the ease with which the features could be used in verbal rules. However, it is important to note that learning was still poorer than in Shepard et al. (1961) and without a direct comparison to the stimuli commonly used in Shepard et al. (1961) replications, the conclusions in terms of the nameability of the stimuli are limited due to other methodological differences.

We argue that Shepard et al.’s (1961) stimuli are specialized even by the standards of the perceptual learning paradigm in that their features can be described in a way that allows extremely compact verbal rules. In the classic stimuli, the noun descriptor of the object as a whole is treated as one of the features, for example, for a small, black triangle the name of the object overall is “triangle”; however, that is also one of the features. This contrasts with the rocket ship stimuli in which “rocket ship” is the name of the object but is not a feature value used to discriminate category instances. Additionally, Shepard et al.’s stimuli are composed of features that refer to the instance as a whole and therefore do not require additional descriptors to discriminate between the feature dimensions. For example, with the size feature dimension, Shepard et al.’s stimuli may have the value “big” and that is sufficient to describe that feature value because it refers to the instance as a whole. With the rocket ship stimuli, the size dimension needs an extra descriptor, “big booster” to indicate what is big. The learning based on Shepard et al.’s stimuli therefore benefits from these advantages that allow rules to be specified very compactly. Experiment 3 directly compared the classic Shepard et al. (1961) stimuli to the rocket ship stimuli used in Experiment 2 in the context of a common methodology.

## Experiment 3

The learning in Experiment 2 was not as good as Nosofsky et al. (1994a), the standard replication of Shepard et al. (1961). We have argued that this is due to the specialized nature of the stimuli used in Shepard et al. (1961) and its replications. Despite both sets of stimuli having the same feature dimensions of color, shape and size from the classic stimuli, the Shepard et al. stimuli allow especially compact rules, for example, “black triangles, white circles group A else group B,” in contrast to the rocket ship stimuli, for example, “wide wings, blue stripe rockets and narrow wings, green stripe rockets thab else lork.”

The purpose of this experiment was to contrast the classic stimuli with the rocket ship stimuli from Experiment 2 on Type II classification learning and compare the lengths of the rules from a qualitative question. We chose Type II as the configural rule is the simplest, non-trivial rule. We did not include feature inference learning because the classic stimuli do not facilitate the feature removability needed for feature inference.

### Materials and methods

#### Participants

Sixty Cardiff University students participated for course credit or payment.

#### Materials and procedure

The materials and procedure were identical to the Type II classification learning condition from Experiment 2 for the rocket ship stimuli condition, except for the removal of the feature inference testing items at the end. The second condition used the stimuli of Shepard et al. (1961), which included category labels (group A and group B) and dimensional variations of shape (triangle/circle), color (black/white), and size (large/small). Note, the size dimension was scaled to be comparable to the overall size of the rocket ship stimuli.

### Results

**Fig. 17:**
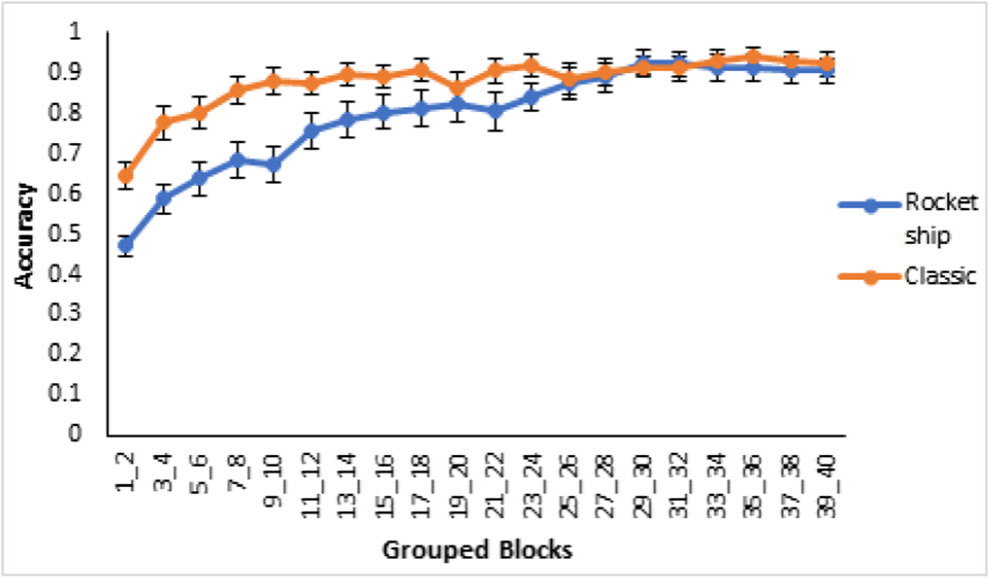
Accuracy as average proportion correct, averaged over two learning blocks for both the rocket ship stimuli condition and the classic stimuli condition. Error bars are standard error

**Fig. 18:**
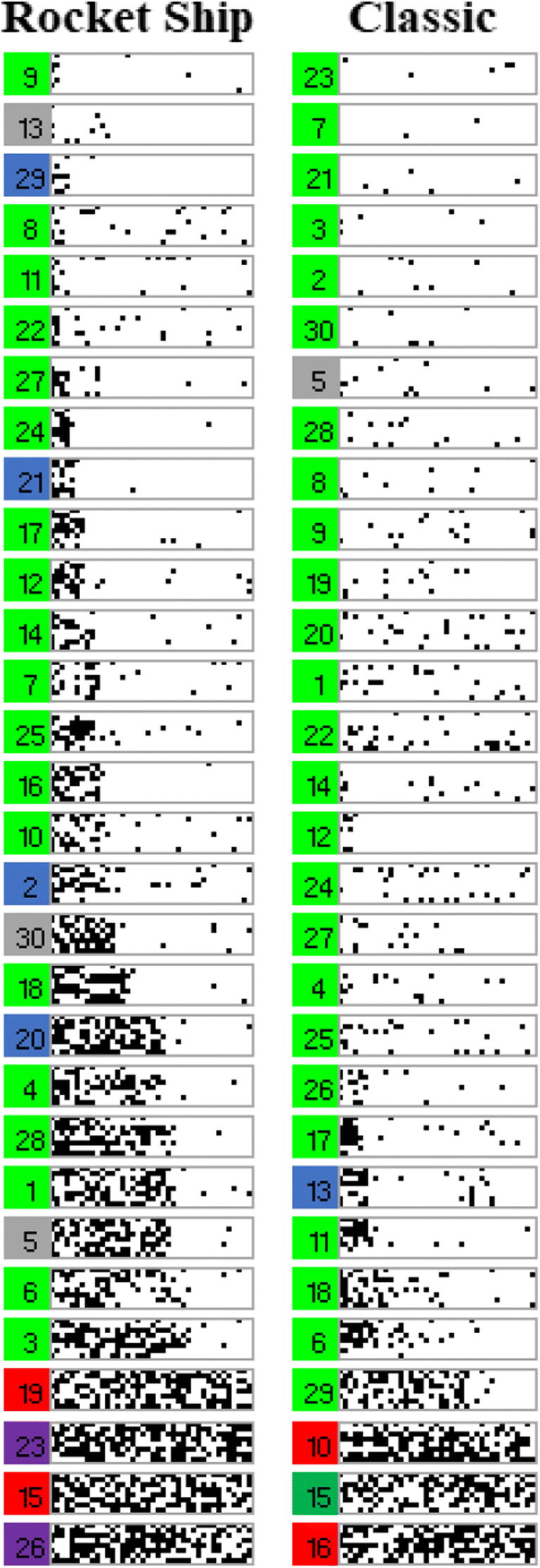
Panels showing the individual performance of each participant in Experiment 3 on every learning trial. White dots indicate a correct answer on an individual trial and black dots indicate an incorrect answer. Each row represents a single category instance, and the instances are ordered as in Table 1. Therefore, each row within a panel shows performance on one specific trial across the 40 learning blocks. Each column of panels represents a learning condition as indicated by the column headers

**Fig. 19:**
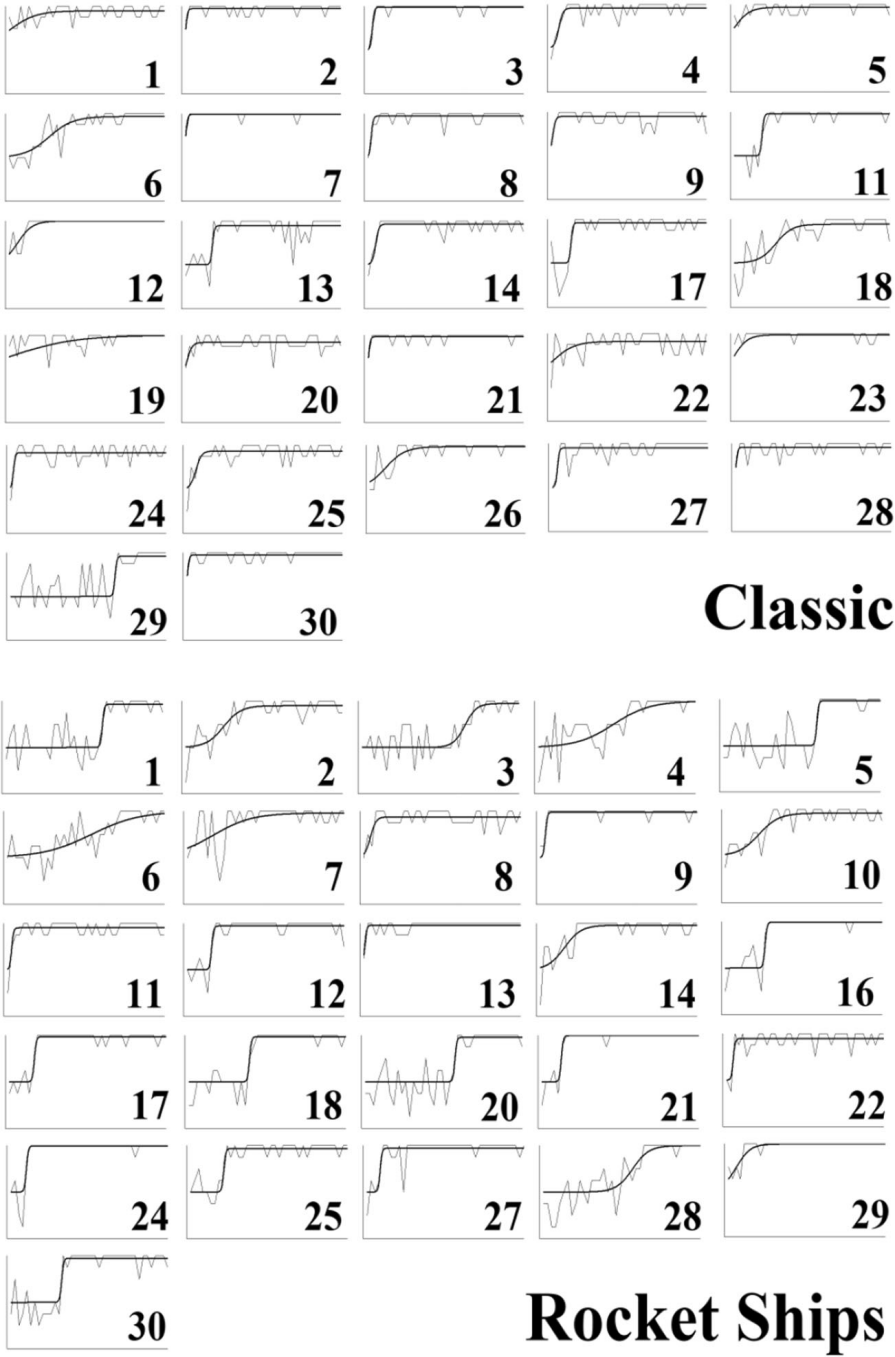
Sigmoid functions fitted to learning curves for participants who showed any learning (in the error diagrams; Fig. 18) in the classic and rocket ship stimuli conditions

For the early learning blocks (1–2; Fig. 17), learning was significantly faster for the classic stimuli than the rocket ship stimuli (*t*(54)=4.1, *p*<0.001, *d*=1.06; bootstrapped confidence interval (0.1, 0.3) and *p*<0.001). Individual error diagrams (Fig. 18) also show this and replicate the rapid transitions from poor performance to high accuracy, indicative of rule use. And this is supported by the fits of sigmoid functions to the individual participant learning curves for participants who showed any learning (Fig. 19).

**Fig. 20:**
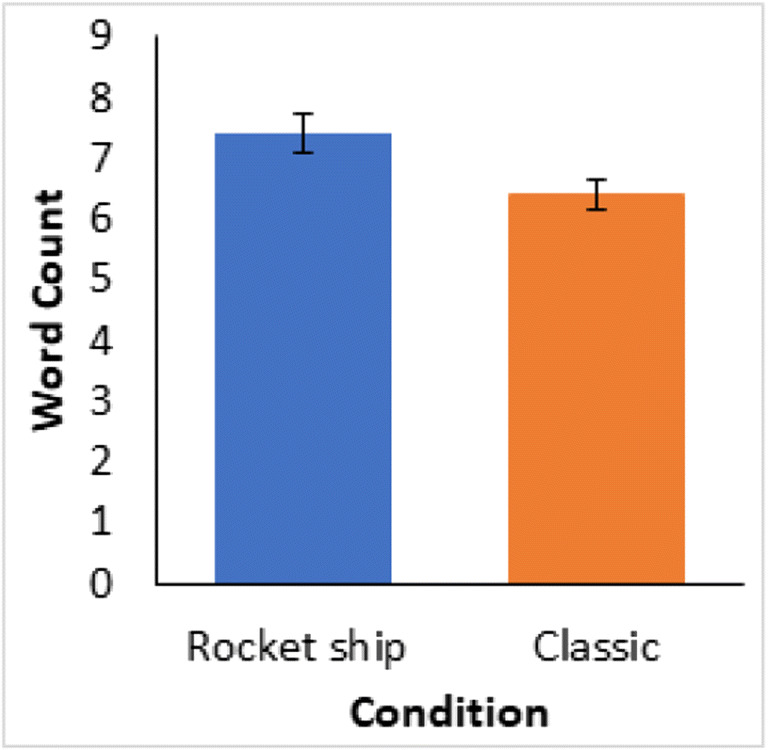
Average word count for the extraction of the specified optimal verbal rule for the participants who achieved the learning criterion of greater than 75% correct over the last four learning blocks. Error bars are standard error

The key rule use query prompted participants to specify how they learned the task. Importantly, participants’ full descriptions generally used more words than necessary to specify a rule and included comments not directly about their rule, for example, one participant stated, “Yes, leaving the mouse cursor on Group A I would select it if either an unfilled circle or filled triangle appeared. Otherwise I selected Group B then reset my cursor on Group A. I focused only on Group A by using true/false methods to switch and select Group B if necessary. Also, I repeated the words, "unfilled circle, filled triangle" in my head.” From this we inferred the rule, “unfilled circle, filled triangle, Group A.” Thus, our data tabulation was in terms of a rule for one category with the other category implied to be instances that did not satisfy this rule, with additional comments and connectives removed, and only words which directly described features and category labels were included. The participants who did not learn were removed. There were significantly fewer words in the tabulated configural rules for the classic stimuli compared to the rocket ship stimuli (Fig. 20) (*t*(41)=2.4, *p*=0.021, *d*=0.74; bootstrapped confidence interval (0.286, 5.190), *p*=0.039).

**Fig. 21:**
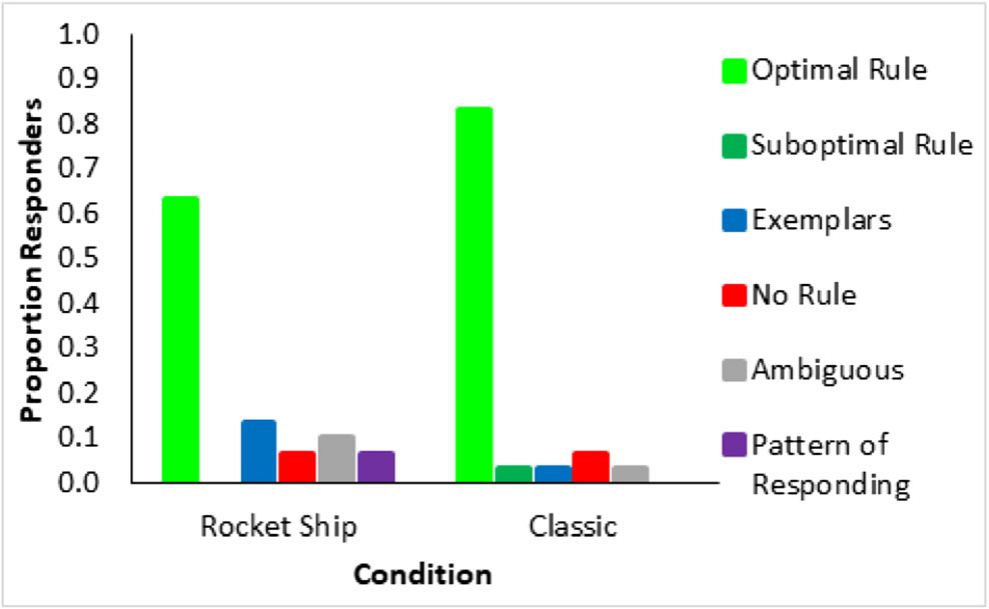
Proportion of all participants who reported using each kind of representation for each learning condition in Experiment 3

Reported rule use (Fig. 21) was high in both conditions with 63% of participants reporting an accurate verbal rule in the rocket ship stimuli condition and, even higher, 83% of participants reporting an accurate rule in the classic stimuli condition. And the somewhat higher rule use for the classic stimuli was consistent with the better learning performance (Fig. 17).

### Discussion

Learning of the classic stimuli was better than the rocket ships, and the classic stimuli were described more compactly in verbal rules. This implies that the especial rule compactness of the classic stimuli influences learning performance of the Shepard et al. (1961) types; compact verbal rules facilitate learning.

## General discussion

Learning about categories by feature inference is plausible given the functional importance of feature inference in categorization. This research compared classification and feature inference learning of four of the classic category structures from Shepard, Hovland, and Jenkins (1961; replicated and evaluated many times; Edmunds & Wills, 2016; Griffiths et al., 2008; Kruschke, 1992; Kurtz, 2007; Lewandowsky, 2011; Love, 2002; Love et al., 2004; Nosofsky et al., 1994a, b; Rehder & Hoffman, 2005; Smith et al., 2004; Žauhar et al., 2016). Our main research question was: Do classification and feature inference learning result in different patterns of learnability across the four types (Types I, II, V, and VI) as a consequence of the presence of the category labels in feature inference? The observed differences between classification and feature inference learning suggest that the answer is a qualified *yes* in terms of support for the label-bias hypothesis, i.e., a bias to try to use label-based rules in feature inference learning in contrast to classification learning. This manifested most directly in terms of Type I being learned faster by feature inference than by classification in Experiments 1 and 2 and by suboptimal rule use in Type V from Experiment 1 where the stimuli were hard to learn. It also manifested as a similar feature inference advantage for early Type II learning in Experiment 2. More subtly, this manifested in terms of less differentiation of the harder types for feature inference learning in contrast to the classic type ordering for classification learning, specifically because Type VI was learned significantly more quickly by feature inference than classification.

Despite the support for the bias hypothesis, the results did not support a distinct kind of representation for classification versus feature inference learning; the results supported the preponderance of verbal rule representation for both in contrast to the conclusions of prior research (Anderson et al., 2002; Johansen & Kruschke, 2005; Yamauchi & Markman, 1998; etc.). Notwithstanding skepticism about self-report data, the qualitative data showed good correspondence between what participants said about rules, what stimuli they saw and how well they learned: 64% of participants who learned in the various conditions of Experiment 2 were able to fully articulate accurate verbal rules. In particular, 21% of the participants who learned Type VI in Experiment 2 explicitly specified the details of the Odd-Even rule, a complex rule to describe. It is not clear why participants would be able to specify such verbal rules if this was not how they did the task. Further, the error diagrams, with all participant errors on individual trials (Figs. 6, 10, and 18), show relatively rapid changes in performance from chance to high accuracy consistent with the sudden acquisition of rules for the majority of participants, formally measured using the slopes of sigmoid function fits to the individual participant learning curves (e.g., Figs. 12 and 19). The testing phase evaluated training instances from both classification and feature inference even though participants were only trained on one or the other. Testing trials showed little difference in accuracy on untrained responses (trials trained in the alternative learning condition) versus trained responses consistent with the use of rules, as a rule can be easily reversed in terms of stimulus and response.

Perhaps the most surprising finding of these experiments is in terms of learnability and the specialized nature of the Shepard et al. stimuli. The contrast in performance between Experiments 1 and 2 supported the preponderance of rule-based representations; the implied greater difficulty of using the verbal rules on the stimuli in Experiment 1, despite the plausibility of the stimuli, corresponded to poorer performance but more suboptimal rule use especially for Type V. Changes to the stimuli to allow more compact verbal rules corresponded to better learning in Experiment 2, also shown by less suboptimal rule use in Type V. Experiment 3 directly compared the classic stimuli to the rocket ship stimuli from Experiment 2 and confirmed the superior learnability of the classic stimuli but also emphasized the unusual compactness of the verbal rules they allow.

Despite the undeniable importance of the Shepard et al. (1961) structures, the substantial evidence for rule representation in these tasks does not sit particularly comfortably with the attributes of real-world categories in that real-world categories are not widely believed to be represented solely by rules, i.e., rules in terms of necessary and sufficient conditions. This contrasts with the evidence for the preponderance of rule-based category representations in the results presented here. But, while these results don’t support a difference in the kind of representation between classification and feature inference learning (Anderson et al., 2002; Johansen & Kruschke, 2005; Yamauchi & Markman, 1998; etc.), the label-bias hypothesis is notably consistent with the spirit of the representational difference hypothesis from Yamauchi and Markman (1998), Anderson et al. (2002), etc. and with the importance of category labels in category based decision making (Gelman & Markman, 1986; Johansen et al., 2015; Yamauchi & Markman, 2000; etc.). Feature inference is plausibly less about the contrast between categories and more focused on the internal attributes of the category as centered on a conceptual label.

## Figures and Tables

**Fig. 22:**
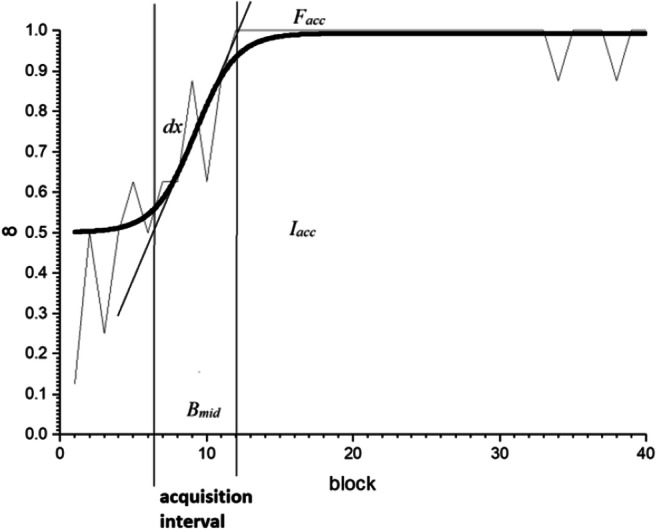
Boltzmann sigmoid function fit as a bold curve to participant 8’s learning curve (accuracy by block) from classification learning of Type II in Experiment 2

**Fig. 23:**
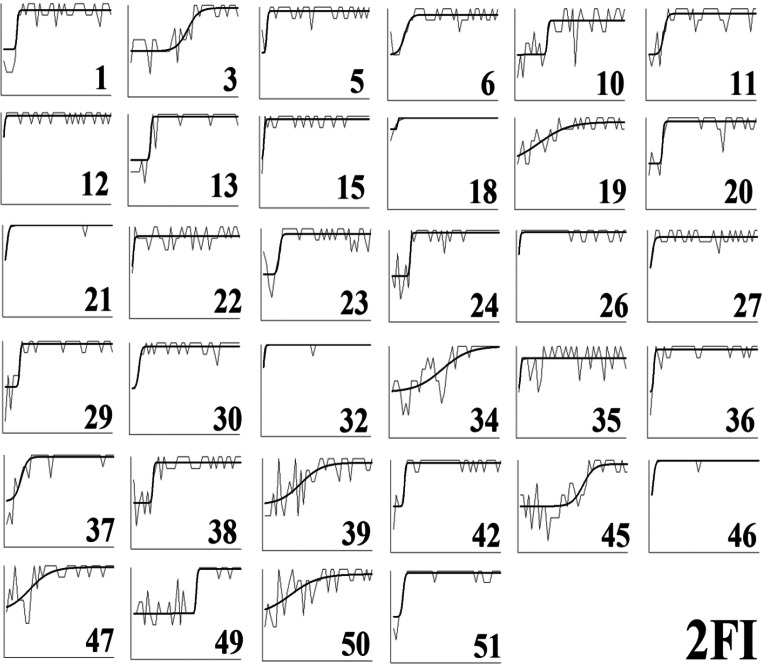
Sigmoid functions fitted to learning curves for participants who showed any learning (in the error diagrams, Fig. 10) in the Type II feature inference condition

## Appendix 1: All Experiment 2 testing trials on the second and third stimulus dimensions.

**Table 2:** Additional testing trials for all types in Experiment 2 on the second and third stimulus dimensions

Testing Trials			
Type I	Type II	Type V	Type VI
A1?1	A1**1**1	A1?1	A1**1**1
A1?1	A1**1**0	A1**1**0	A0**1**0
A1?0	A0**0**1	A1**?**1	A0**0**1
A1?0	A0**0**0	A0**0**0	A1**0**0
B0?1	B0**1**1	B0?1	B0**1**1
B0?1	B0**1**0	B0?1	B1**1**0
B0?0	B1**0**1	B0**1**0	B1**0**1
B0?0	B1**0**0	B1**0**0	B0**0**0
A11?	A11?	A11?	A11**1**
A10?	A11?	A11?	A11**0**
A11?	A00?	A10**1**	A00**1**
A10?	A00?	A00**0**	A10**0**
B01?	B01?	B01?	B01**1**
B00?	B01?	B00**1**	B11**0**
B01?	B10?	B01?	B10**1**
B00?	B10?	B10**0**	B00**0**

*Note.* “A” and “B” refer to the two category labels and the subsequent three numbers refer to the three binary-valued feature dimensions and the feature values on those dimensions. Bolded features and question marks indicate what was queried for a given instance in a given condition. Bold features represent a correct answer and question marks indicate the lack of an unambiguous correct answer

## Appendix 2: Coding of reported qualitative strategy results.

Based on responses to the qualitative learning strategy question at the end of Experiments 2 and 3, participants were coded into one of seven groups: optimal rules, suboptimal rules, no rule/poor reported learning, prototypes, a pattern of responding, memorized exemplars or ambiguous. The “Optimal rules” group coding used a fairly harsh criteria of needing to not only fully state a rule but also that that rule needed to be accurate given the stimulus assignments they actually saw. For Type I the optimal rule was a first dimension, unidimensional rule, for Type II it was a configural rule based on the first two dimensions, for Type V it was a unidimensional rule with two, full instance exceptions and for Type VI it was the Odd-Even rule. “Suboptimal rules” were coded as any specification of a rule that was not the optimal rule for the condition a participant was in e.g. a unidimensional rule in Type II. Participants were coded into the “No rule/poor reported learning” group if they indicated that they had not been able to learn the task. The “Exemplars” group was based on a more relaxed coding in terms of a mention of an attempt to use an instance memorization strategy or, alternatively, a specification of more than two individual instances (so as to distinguish this from optimal rule users in Type V specifying exceptions). Participants were coded into the “Ambiguous” group if they were unclear and/or gave responses that lacked sufficient information. A “prototype” coding required three or more features to be described as “mostly” belonging to one category. Finally, a “pattern of responding” group was coded as a strategy based on the order of responses across trials rather than the stimuli, e.g., alternating between category A and B across a series of trials regardless of the specific stimuli.

## Appendix 3: Procedures for fitting Sigmoid functions to individual learning curves.

We used the graphing and modeling package Origin to fit Boltzmann sigmoid functions to all individual participant learning curves in all conditions of Experiments 2 and 3. The Boltzmann sigmoid, e.g., fitted to participant 8 from classification learning of Type II in Fig. 22, is specified by Equation 1. The Boltzmann predicts accuracy, a_cc_, as a function of learning block, b_i_, using four parameters: The first is an initial accuracy parameter for the start of learning before performance started to improve, I_acc_, and this was fixed at 0.5, e.g., Fig. 22. The second was a final accuracy parameter, F_acc_, corresponding to the level of performance beyond which no substantive improvement occurred. This was a free parameter constrained to be between 0.5 and 1 as not all participants achieved perfect accuracy (though participant 8 in Fig. 22 was very close). The third parameter was the center of the threshold part of the function in terms of block, B_mid_ (treated as a continuous variable), corresponding to the location of most rapid improvement in performance and constrained to be a value between 1 and 40 (as determined by 40 learning blocks total). And the fourth parameter was the “time constant parameter,” dx, that is related to the slope of the threshold by Equation 1b and was constrained to be between 0.25 and 100. The constraints on the time constant parameter were derived from specifying the slopes corresponding to the slowest and fastest possible rates of acquisition over 40 blocks. Origin adjusted the free parameters within the specified ranges to minimize the discrepancy between the function’s predicted accuracy and participants’ actual accuracy over all learning blocks. Finally, we used a straight line based on the slope through the midpoint, Equation 1b, to infer the acquisition interval, e.g. the two vertical lines in Fig. 22, as the difference between the two points where the diagonal slope line intersected the straight lines corresponding to initial, I_acc_ , and final, F_acc_, performance.

1 $$ {a}_{cc}=\frac{I_{acc}-{F}_{acc}}{1+{e}^{\frac{\left({b}_i-{B}_{mid}\right)}{dx}}}+{F}_{acc} $$

1b $$ {Sl}_{mid}=\frac{F_{acc}-{I}_{acc}}{4\cdotp dx} $$
